# Glucose-6-phosphatase catalytic subunit 2 negatively regulates glucose oxidation and insulin secretion in pancreatic β-cells

**DOI:** 10.1016/j.jbc.2022.101729

**Published:** 2022-02-15

**Authors:** Mohsin Rahim, Arya Y. Nakhe, Deveena R. Banerjee, Emily M. Overway, Karin J. Bosma, Jonah C. Rosch, James K. Oeser, Bo Wang, Ethan S. Lippmann, David A. Jacobson, Richard M. O'Brien, Jamey D. Young

**Affiliations:** 1 Department of Chemical and Biomolecular Engineering, Vanderbilt University, Nashville, Tennessee, USA; 2 Department of Molecular Physiology and Biophysics, Vanderbilt University, Nashville, Tennessee, USA; 3 Department of Biomedical Engineering, Vanderbilt University, Nashville, Tennessee, USA; 4 Department of Neurology, Vanderbilt University, Nashville, Tennessee, USA

**Keywords:** metabolic flux analysis, glucose uptake, insulin secretion, systems biology, glycolysis, G6PC2, α-KG, α-ketoglutarate, CAC, citric acid cycle, CAM, cardiovascular-associated mortality, ER, endoplasmic reticulum, FACS, fluorescence-activated cell sorting, FBG, fasting blood glucose, G6P, glucose-6-phosphate, G6PC, glucose-6-phosphatase catalytic subunit, GSIS, glucose-stimulated insulin secretion, IDH, isocitrate dehydrogenase, KO, knockout, MFA, metabolic flux analysis, MID, mass isotopomer distribution, T2D, type 2 diabetes, WT, wild-type

## Abstract

Elevated fasting blood glucose (FBG) is associated with increased risks of developing type 2 diabetes (T2D) and cardiovascular-associated mortality. *G6PC2* is predominantly expressed in islets, encodes a glucose-6-phosphatase catalytic subunit that converts glucose-6-phosphate (G6P) to glucose, and has been linked with variations in FBG in genome-wide association studies. Deletion of *G6pc2* in mice has been shown to lower FBG without affecting fasting plasma insulin levels *in vivo*. At 5 mM glucose, pancreatic islets from *G6pc2* knockout (KO) mice exhibit no glucose cycling, increased glycolytic flux, and enhanced glucose-stimulated insulin secretion (GSIS). However, the broader effects of *G6pc2* KO on β-cell metabolism and redox regulation are unknown. Here we used CRISPR/Cas9 gene editing and metabolic flux analysis in βTC3 cells, a murine pancreatic β-cell line, to examine the role of *G6pc2* in regulating glycolytic and mitochondrial fluxes. We found that deletion of *G6pc2* led to ∼60% increases in glycolytic and citric acid cycle (CAC) fluxes at both 5 and 11 mM glucose concentrations. Furthermore, intracellular insulin content and GSIS were enhanced by approximately two-fold, along with increased cytosolic redox potential and reductive carboxylation flux. Normalization of fluxes relative to net glucose uptake revealed upregulation in two NADPH-producing pathways in the CAC. These results demonstrate that *G6pc2* regulates GSIS by modulating not only glycolysis but also, independently, citric acid cycle activity in β-cells. Overall, our findings implicate G6PC2 as a potential therapeutic target for enhancing insulin secretion and lowering FBG, which could benefit individuals with prediabetes, T2D, and obesity.

Glucose-6-phosphatase, a multicomponent system located in the endoplasmic reticulum (ER), catalyzes the conversion of glucose-6-phosphate (G6P) to glucose (1). This enzyme system is composed of several integral membrane proteins including a G6P transporter, encoded by *SLC37A4*, which carries the substrate from the cytosol to the ER lumen. Once in the ER, G6P is hydrolyzed by a glucose-6-phosphatase catalytic subunit (G6PC) to glucose and inorganic phosphate, and these products are subsequently transported to the cytosol. Three G6PC isoforms, encoded by *G6PC1*, G*6PC2*, and *G6PC3*, have been identified and are selectively expressed in different tissues (1, 2). *G6PC1* is predominantly expressed in the liver and kidneys where it catalyzes the terminal step in endogenous glucose production through gluconeogenesis and glycogenolysis. *G6PC3* is highly expressed in the kidneys, testis, skeletal muscle, and brain (1) where it functions to eliminate the noncanonical metabolite, 1,5-anhydroglucitol-6-phosphate (3). *G6PC2*, also known as the islet-specific glucose-6-phosphatase catalytic subunit-related protein (IGRP), is predominantly expressed in pancreatic islet β-cells (1).

Previous work from our group has shown that G6PC2, along with glucokinase (GK), forms a futile substrate cycle where G6PC2 dephosphorylates G6P, generated by GK, back into glucose. This suggests that G6PC2, in conjunction with GK, may regulate glycolytic flux and consequently affect the glucose sensitivity of glucose-stimulated insulin secretion (GSIS) (4). Various observations support this model. Glucose-6-phosphatase activity (4) and glucose cycling (5) are abolished in *G6pc2* knockout (KO) islets, *G6pc2* KO mice exhibit reduced fasting blood glucose (FBG) with no change in fasting plasma insulin (FPI) compared with wild-type (WT) controls as a consequence of a leftward shift in the dose–response curve for GSIS (4). *G6pc2* KO islets incubated for short durations (<2 h) in submaximal glucose also show higher glycolytic flux and increased GSIS compared with islets isolated from WT littermates (2, 4). These prior results suggest that G6PC2 negatively regulates GSIS by opposing flux through glycolysis, but the effect of *G6pc2* deletion on other downstream pathways that control insulin release, such as the citric acid cycle (CAC) and pentose phosphate pathway (PPP), is unknown. Genetic studies in humans have generated data that indicate G6PC2 may regulate pulsatile insulin secretion (6), which suggests that G6PC2 may regulate aspects of β-cell metabolism other than glycolysis.

To better understand the role of *G6pc2* in β-cells, we describe here the application of ^13^C metabolic flux analysis (MFA) to assess global changes in glucose and oxidative metabolism in response to *G6pc2* deletion. MFA is a stable isotope-based approach that relies on the inherent assumption that the system under investigation is at metabolic steady state. However, obtaining sufficient isotope enrichment to enable precise flux estimation in cell culture systems often requires extended incubation times (≥24 h) (7). Unfortunately, prolonged culture of primary islets causes gradual loss of their *in vivo* metabolic phenotype (5), which may explain why we observed a difference in glucose cycling but not in net glucose uptake or media insulin concentration between WT and *G6pc2* KO islets (8). To minimize the confounding effects of phenotypic instability and cellular heterogeneity that complicate *ex vivo* studies of primary islets, we used a pancreatic mouse β-cell line (βTC3) to examine the metabolic effects of *G6pc2* loss.

We utilized CRISPR-Cas9 gene editing to generate stable G6pc2 KO and WT βTC3 cell lines for MFA studies. We also developed a mathematical model to assess isotope labeling measurements and quantify flux through the major pathways of glycolysis, PPP, CAC, and anaplerosis in β-cells. Metabolic fluxes were estimated by simultaneously regressing isotope enrichment measurements from 21 unique metabolite fragment ions and 13 extracellular uptake and excretion rates obtained from WT and KO cells incubated with ^13^C isotopes. The results indicate that *G6pc2* deletion leads to a significant increase in oxidative fluxes and GSIS. An approximate 60% increase in glycolytic and CAC activity was accompanied by an approximately two-fold upregulation in intracellular insulin content and GSIS. Furthermore, we observed an increase in the cytosolic NADPH:NADP^+^ ratio along with a two-fold flux increase through the reductive carboxylation pathway from glutamine to citrate. Importantly, normalization of fluxes to the net glucose uptake rate showed increased flux through two NADPH-producing pathways in the CAC, independent of elevated glycolytic flux. These results suggest that G6PC2 also regulates CAC pathways, separate from its modulation of glycolysis. More broadly, these data suggest that G6PC2 could be a potential target for enhancing insulin secretion as well as lowering FBG in individuals with prediabetes, type 2 diabetes (T2D) and obesity.

## Results

### βTC3 cell line is a representative *in vitro* model to study the effects of G6PC2 on β-cell metabolism

**Figure 1:**
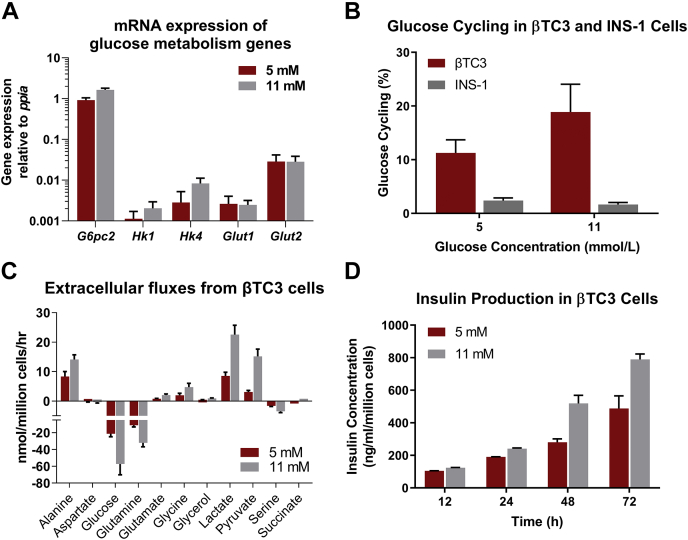
**Validation of βTC3 cell line as a representative model to study metabolic regulation by *G6pc2*.***A*, mRNA expression of genes regulating glucose metabolism. Data represent means ± SEM (n = 3) relative to expression of the housekeeping gene *ppia*. *B*, estimated percentage glucose cycling in βTC3 and INS-1 cells at 5 and 11 mM glucose concentrations. Data represent means ± SEM (n = 3). *C*, extracellular uptake and excretion rates measured in βTC3 cells incubated at 5 and 11 mM glucose concentrations. Positive values represent net excretion while negative values indicate net uptake. Data represent means ± SEM (n = 3). *D*, media insulin content over time for βTC3 cells incubated at 5 and 11 mM glucose concentrations. Data represent means ± SEM (n = 3).

Prior to conducting metabolic studies on βTC3 cells, we validated that the cell line has characteristics suitable for studying the function of G6PC2. We examined expression of *G6pc2*, *Gck* (HK IV), and *Slc2a2*. *Gck* and *Slc2a2* encode glucokinase and the GLUT2 glucose transporter, respectively (9). All three genes were expressed in βTC3 cells, and their expression was not dependent on glucose concentrations (Fig. 1*A*). We also verified that glucose cycling (Figs. 1*B* and S1, *A*, *C*, and *E*), a functional readout of G6PC2 activity, was present at levels comparable to those previously measured in primary islets at 5 mM (∼10%) and 11 mM (∼20%) glucose concentrations (8, 10). In contrast, glucose cycling studies in INS1-832/13 cells (Figs. 1*B* and S1, *B*, *D*, and *F*), a rat insulinoma cell line, showed negligible glucose cycling (≤3%) and net release of glucose (Fig. S1*F*, ∼1 nmol/10^6^ cells/hr), further validating that *G6pc2* is a pseudogene in rats (11). Lastly, uptake and excretion rates of metabolites connected to central carbon metabolism roughly doubled when the glucose concentration was increased from 5 to 11 mM (Fig. 1*C*), and this was associated with a linear increase in insulin secretion over 72 h (Fig. 1*D*). These results indicate that β-cell-specific features are conserved within βTC3 cells, making the cell line a representative model for our studies investigating the effects of G6PC2 on metabolism.

### Generation and validation of βTC3 G6pc2 knockout cells

**Figure 2:**
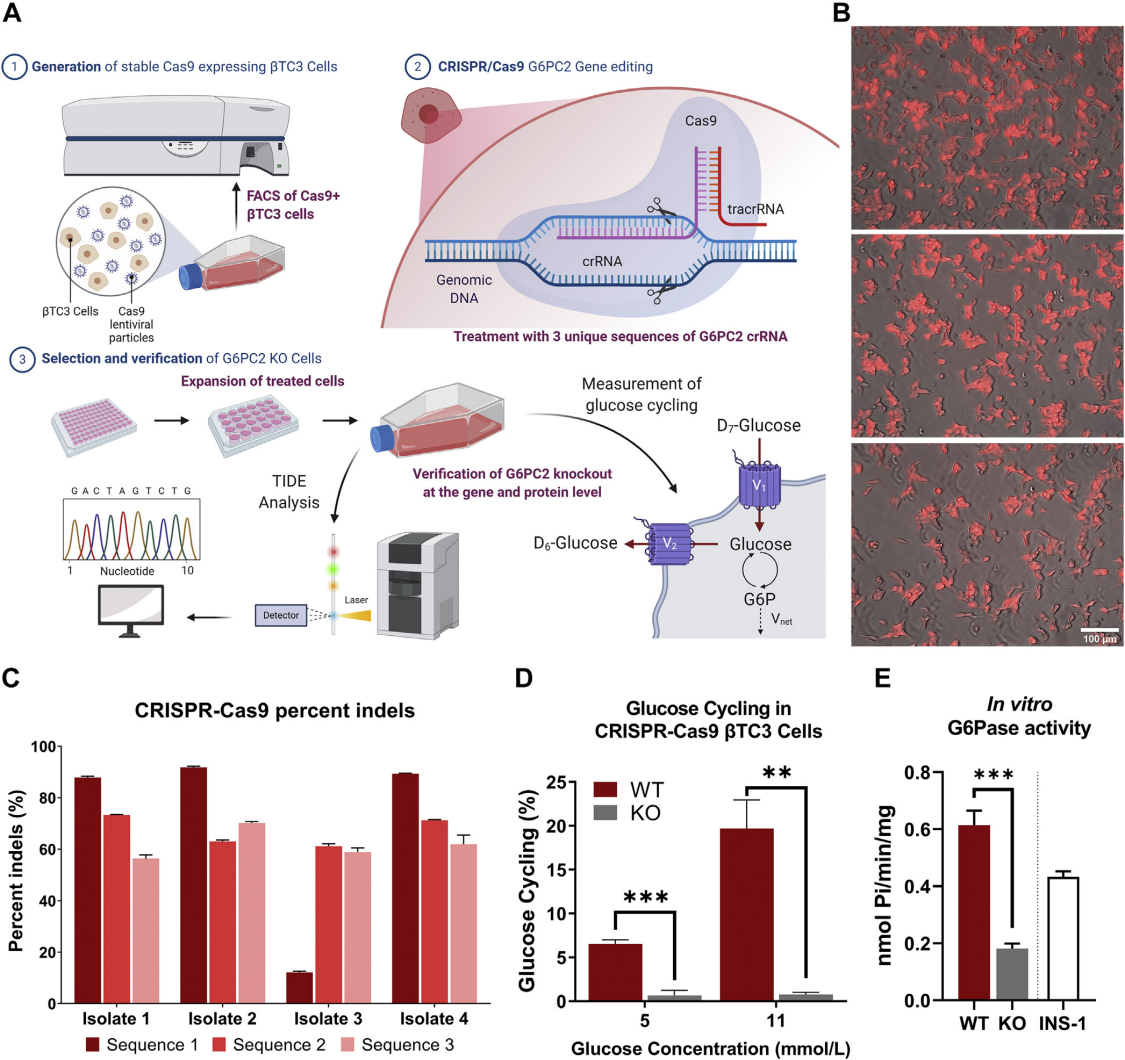
**Generation of βTC3 *G6pc2* knockout and control cell lines using CRISPR-Cas9.***A*, schematic providing an overview of βTC3 *G6pc2* KO cell line development using CRISPR-Cas9 (See Experimental procedures for details). *B*, mKate2 and brightfield superimposed images from three biological replicates of βTC3 cells expressing Cas9 protein. The single cell clone with the highest mKate2 fluorescence, shown here, was selected for CRISPR RNA transfection. *C*, TIDE analysis showing the total percentage of insertions and deletions (indels) around three different loci targeted with three different CRISPR RNA sequences in four biological isolates of the single cell clone shown in panel (*B*). *D*, verification of loss of *G6pc2* functional activity in KO cells by measurement of glucose cycling (see Ref. (7) for details) at 5 and 11 mM glucose concentrations. Cas9 expressing single cell clone from panel (*B*) was used as βTC3 wild-type (WT) control. Data represent means ± SEM, ∗∗∗*p* < 0.01, ∗∗*p* < 0.05 (n = 3). *E*, measurement of glucose-6-phosphatase (G6Pase) activity *in vitro* to further validate the knockout of *G6pc2* in βTC3 cells. G6Pase activity from INS-1 cells, which do not express functional G6PC2, is included as a background control. Values represent the release of inorganic phosphates per min per mg of total protein. Data represent means ± SEM, ∗∗∗*p* < 0.01 (n = 3). KO, knockout.

To examine the effects of *G6pc2* deletion in βTC3 cells, we engineered *G6pc2* knockout (KO) and control (WT) cell lines by using the CRISPR/Cas9 gene editing technique. We first generated a βTC3 cell line with constitutive Cas9 expression using lentiviral transduction (Fig. 2*A*). The highest Cas9-expressing βTC3 single cell clones were identified using fluorescence-activated cell sorting (FACS) followed by immunofluorescent imaging (Fig. 2*B*). To generate KO cells, three unique synthetic gRNAs (crRNA:tracerRNA conjugates, each targeting a specific cut site) were delivered to the highest Cas9-expressing single cell clone using lipofection. To generate WT control cells, only tracerRNA was delivered to the highest Cas9 expressing single cell clone using the same transfection protocol. After three rounds of gRNA or tracerRNA treatment, four biological isolates were expanded and analyzed using the Tracking of Indels by DEcomposition (TIDE) genomic assessment tool to quantify the percentage knockout (see Experimental procedures for details). Computational assessment of Sanger-sequenced genomic DNA showed all three cut sites were predicted to have indel formations with biological isolates 2 and 4 having >90% indel formation at the first cut site (Fig. 2*C*), which is indicative of highly efficient knockout. To confirm loss of G6PC2 functional activity, we measured glucose cycling in KO isolates 2 and 4. Our results indicate that glucose cycling was almost completely abolished (Fig. 2*D*) with negligible glucose release flux (Fig. S2*D*) in the KO cells at 5 and 11 mM glucose concentrations. Additionally, there was no change in the cycled abundance of glucose over time in either KO isolate, whereas an 8 to 10% increase was observed in WT isolates within 24 h of incubation (Fig. S2, *A* and *B*). The total glucose uptake flux (Fig. S2*C*) trended higher in the KO isolates; however, the differences were not significant. Furthermore, microsomes isolated from KO cells showed significant downregulation of G6Pase activity *in vitro* relative to WT cells (Fig. 2*E*), validating the loss of functional G6PC2 expression in KO cells.

**Figure 3:**
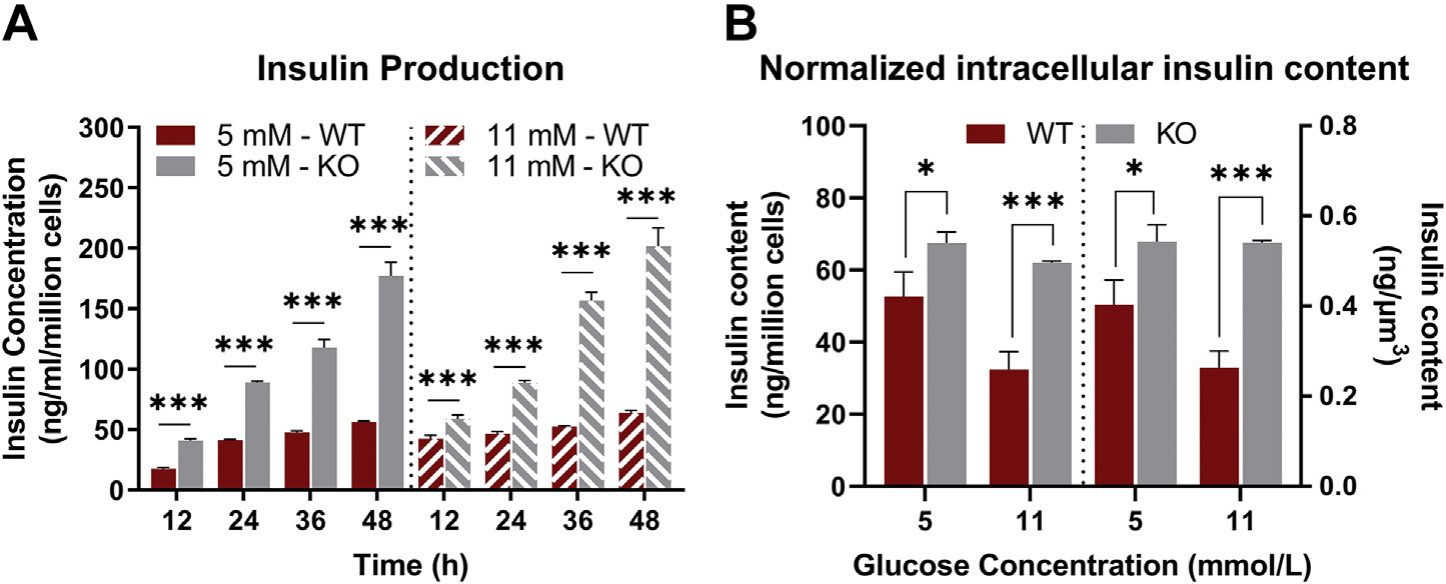
**Knockout of *G6pc2* in βTC3 cells leads to increased GSIS.***A*, insulin production normalized to cell count at 5 mM (*left*) and 11 mM (*right*) glucose concentrations in βTC3 *G6pc2* WT and KO cells. Data represent means ± SEM, ∗∗∗*p* < 0.01, ∗∗*p* < 0.05 (n = 4). *B*, intracellular insulin content normalized to cell count (*left*) and to total surface area (*right*) at 5 and 11 mM glucose concentrations in βTC3 *G6pc2* WT and KO cells. Data represent means ± SEM, ∗∗∗*p* < 0.01, ∗*p* < 0.10 (n = 4). GSIS, glucose-stimulated insulin secretion; KO, knockout.

To further validate our novel cell model, we measured intracellular and media insulin concentration in *G6pc2* WT and KO cells at 5 and 11 mM glucose concentrations. Contrary to primary islets, where longer incubation times (>24 h) led to normalization of GSIS in KO cells due to metabolic adaptation (8), we saw a consistent elevation of media insulin production by βTC3 KO cells compared with WT cells at both glucose concentrations examined (Fig. 3*A*). Measurement of intracellular insulin content also showed that KO cells not only secreted more insulin but also stored more insulin per cell and per cell surface area (Fig. 3*B*). Collectively, these results demonstrate that the engineered βTC3 KO cell clones had negligible G6PC2 activity and therefore provided a cellular model of *G6pc2* deletion. These results also further support our previously published results characterizing G6PC2 as a negative regulator of GSIS (4).

### Assessment of metabolic fluxes reveals increased oxidative metabolism due to loss of G6PC2

**Figure 4:**
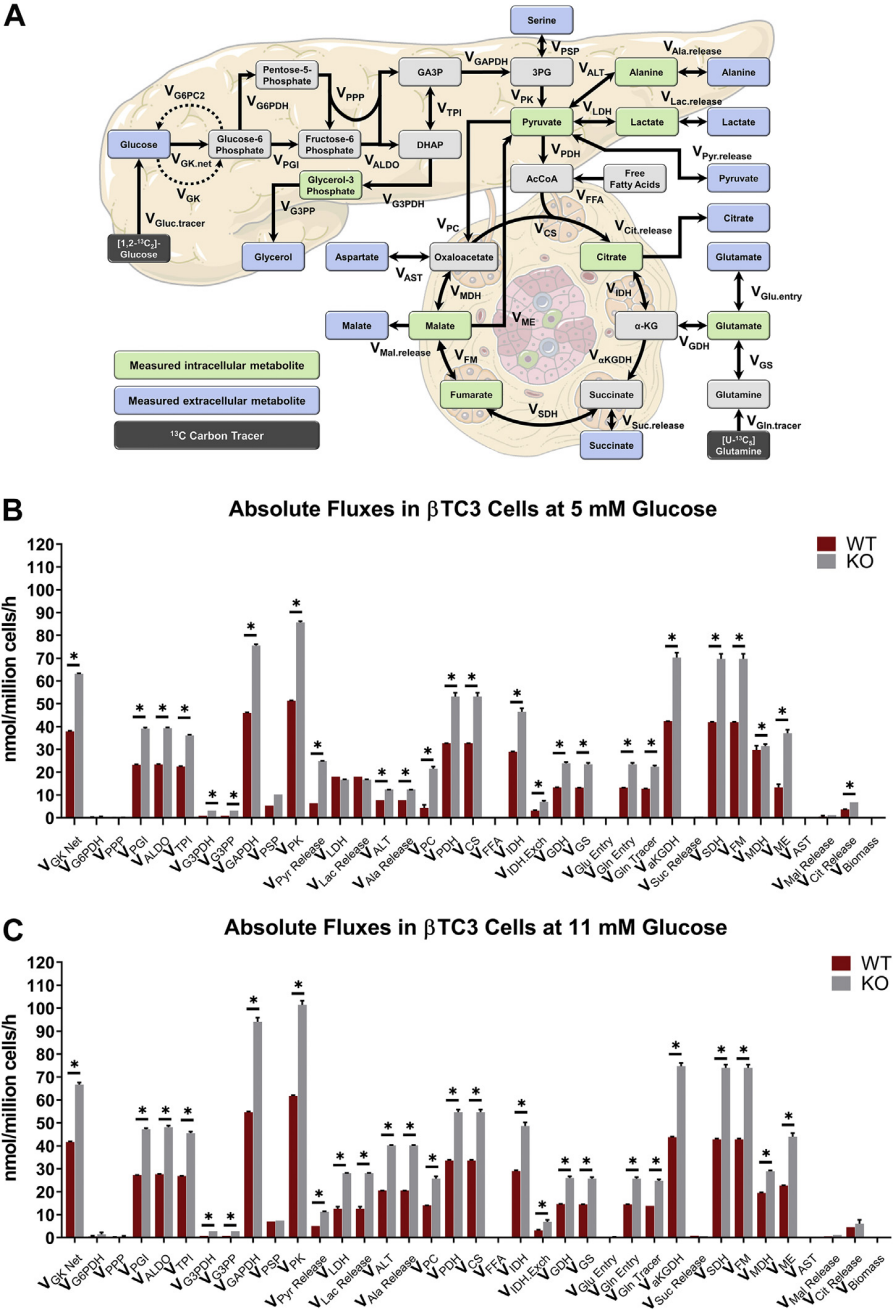
**MFA in βTC3 *G6pc2* WT and KO cells shows increased absolute flux through glycolytic and mitochondrial pathways.***A*, flux network representing oxidative βTC3 metabolism constructed in INCA (58, 59). Measured media metabolites are shown in *blue* while measured intracellular metabolites are highlighted in *green*. *B*, absolute extra- and intracellular fluxes in *G6pc2* WT and KO βTC3 cells at 5 mM glucose concentration estimated using MFA. Data represent means ± SEM, ∗*p* < 0.05 (n = 3). *C*, absolute extra- and intracellular fluxes in *G6pc2* WT and KO βTC3 cells at 11 mM glucose concentration estimated using MFA. Data represent means ± SEM, ∗*p* < 0.05 (n = 3). KO, knockout; MFA, metabolic flux analysis.

To elucidate the underlying mechanisms responsible for increased GSIS in *G6pc2* KO cells, we developed a β-cell specific mathematical model to determine metabolic fluxes from ^13^C labeling measurements. Stable isotope studies were initially conducted on parental βTC3 cells to determine the optimal incubation times required to reach isotopic steady state using different tracers. These studies showed that glycolytic metabolites reached isotopic steady state within 24 h of incubation with [U-^13^C_6_]glucose (Fig. S3*A*), whereas CAC metabolites did not reach isotopic steady state even after 72 h of labeling (Fig. S3*B*). Labeling with [U-^13^C_5_]glutamine, on the other hand, achieved isotopic steady-state ^13^C enrichment of CAC metabolites between 12 and 24 h of incubation (Fig. S3*C*). Based on these data, we determined that parallel labelling experiments with [1,2-^13^C_2_]glucose and [U-^13^C_5_]glutamine for 24 h would provide an optimal experimental design for steady-state MFA. These tracers were previously shown to produce measurements that are highly sensitivity to fluxes in glycolysis/PPP and CAC, respectively (12). A metabolic model including glycolysis and the oxidative PPP was developed (Fig. 4*A* and Table S1) using previously established carbon atom transitions (12). CAC metabolism was modeled using both oxidative and reductive pathways (Fig. 4*A* and Table S1), the latter of which has been shown to promote GSIS in rat islets (13). In addition to measurements of extracellular uptake and excretion rates (Fig. S4), measured mass isotopomer distributions (MIDs) of several media and intracellular metabolites (Figs. S5 and S6) were also provided as inputs to the metabolic model in order to estimate intracellular fluxes using MFA (see Experimental procedures for details).

Glucose and glutamine were metabolized largely *via* the mitochondrial CAC (Fig. 4, *B* and *C*). Excretion of overflow products (lactate, pyruvate, alanine, and citrate) represented a minor fate for disposal of excess carbon (Figs. 4, *B* and *C* and S4). Negligible fluxes were detected through oxidative PPP and pathways leading to the overflow products aspartate, glycerol, malate, and succinate. Glycolytic and mitochondrial fluxes were substantially elevated in *G6pc2* KO cells compared with WT cells at both glucose concentrations examined. At 5 mM glucose, glycolytic (V_PK_) and mitochondrial (V_CS_) fluxes were ∼67% and ∼62% higher compared with WT cells (Fig. 4*B*), respectively. Increased oxidative fluxes were accompanied by a ∼92% and ∼78% increase in serine catabolism (V_PSP_) and glutamine anaplerosis (V_Gln.entry_), respectively. Additionally, we observed significant increases in the excretion fluxes of pyruvate, alanine, and citrate, but not lactate (Figs. 4*B* and S4*A*). We observed similar increases in oxidative fluxes at 11 mM glucose, with glycolytic (V_PK_) and CAC (V_CS_) fluxes increased by ∼60% and 56%, respectively, compared with WT cells (Fig. 4*C*). Anaplerosis from glutamine, but not serine catabolism, was ∼78% higher in KO cells compared with WT cells. We also observed significant increases in the excretion fluxes of pyruvate, alanine, and lactate, but not citrate (Fig. 4*C*).

**Figure 5:**
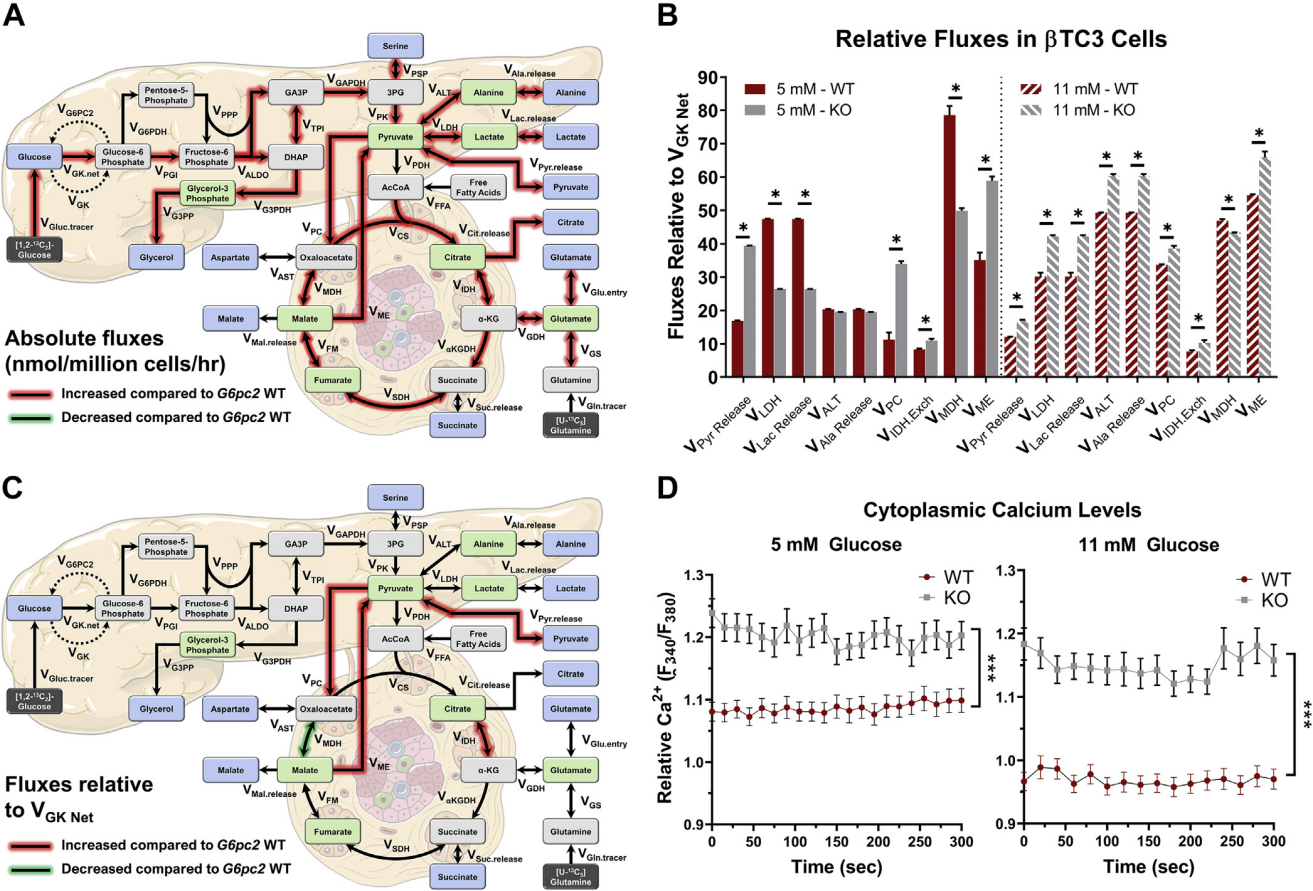
**Metabolic fluxes relative to net glucose uptake reveal flux rerouting through NADPH-producing reactions.***A*, flux map highlighting the absolute metabolic changes associated with *G6pc2* KO in βTC3 cells. Fluxes highlighted in *red* are upregulated compared to *G6pc2* WT βTC3 cells. *B*, subset of selected metabolic fluxes normalized to net glucose uptake (V_GK Net_) in *G6pc2* WT and KO βTC3 cells at 5 mM (*left*) and 11 mM (*right*) glucose concentrations estimated using MFA. Data represent means ± SEM, ∗*p* < 0.05 (n = 3). *C*, flux map highlighting the metabolic changes relative to V_GK Net_ associated with *G6pc2* KO in βTC3 cells. Fluxes highlighted in *red* are upregulated compared with *G6pc2* WT βTC3 cells. *D*, cytoplasmic calcium levels at 5 and 11 mM glucose concentration measured in *G6pc2* WT and KO βTC3 cells. Results are the mean 245 measurements from WT cells and 256 measurements from KO cells taken at 5-s intervals for a total of 300 s. ∗∗∗*p* < 0.01 (n = 3). KO, knockout.

While we saw a significant elevation in absolute glycolytic and mitochondrial fluxes in KO cells (Fig. 5*A*), it was important to determine whether there were metabolic effects arising from the loss of G6PC2 that were independent of increased glycolysis. To accomplish this, we normalized all metabolic fluxes to the net glucokinase flux (V_GK Net_). This normalization revealed more subtle changes in the relative distribution of fluxes within KO cells. In particular, relative fluxes through pyruvate carboxylase (V_PC_/V_GK Net_) and malic enzyme (V_ME_/V_GK Net_) were increased in combination with a decrease in V_MDH_/V_GK_ at both glucose concentrations (Figs. 5, *B* and *C* and S7, *A* and *B*). In addition, we observed a shift in overflow products from increased pyruvate excretion at 5 mM glucose concentration, to increased pyruvate, lactate, and alanine release at 11 mM glucose concentration, potentially due to a shift in the redox state of the KO cells (Fig. 5*B*).

Interestingly, this normalization relative to net glucokinase flux also showed that the exchange flux between citrate and α-ketoglutarate (α-KG) was significantly elevated at both glucose concentrations, suggesting increased reductive activity of isocitrate dehydrogenase (IDH). Reductive carboxylation flux through mitochondrial IDH2 has been proposed as a mechanism that drives NADPH production by cytosolic IDH1, which acts as a coupling factor to regulate insulin secretion (13). While our analysis cannot distinguish contributions from cytosolic and mitochondrial isoforms to the total IDH exchange flux, increased cycling between citrate and α-KG is consistent with enhanced redox coupling between IDH1/2 in KO cells (Fig. 5*C*). Furthermore, similar to data obtained in primary KO islets (4), we measured a significant elevation in cytoplasmic calcium levels within the KO cells at 5 and 11 mM glucose concentrations (Fig. 5*D*). Overall, these results show that deletion of *G6pc2* leads to a broad acceleration in glycolytic and mitochondrial metabolism, primarily by rerouting more glucose and glutamine toward oxidative pathways that supply ATP and redox cofactors linked to GSIS.

### Knockout of G6pc2 promotes a reduced cytosolic redox potential in βTC3 cells

**Figure 6:**
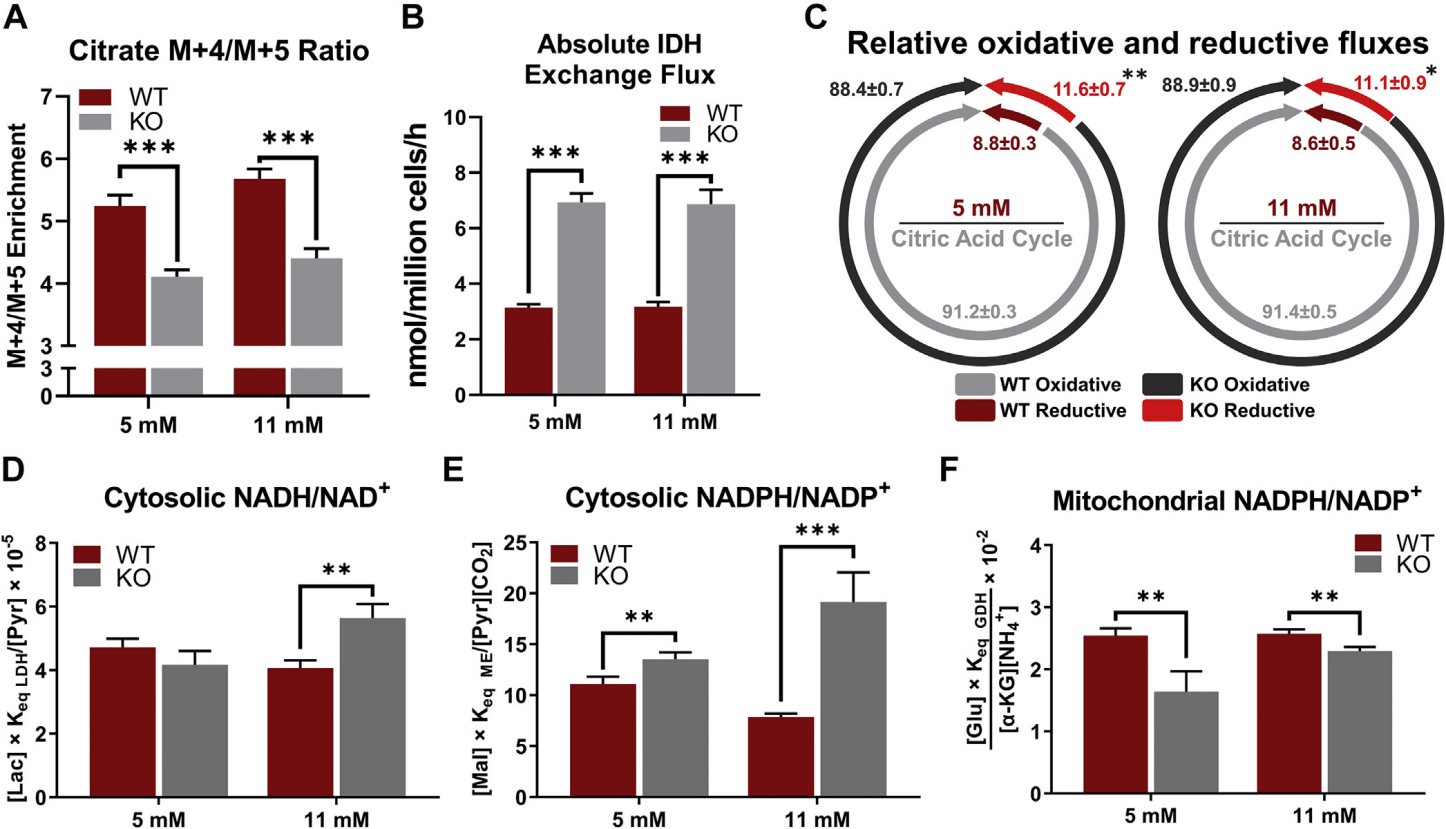
**Knockout of *G6pc2* promotes a reduced redox potential in βTC3 cells.***A*, ratio of M + 4/M + 5 isotopomer enrichment in *G6pc2* WT and KO βTC3 cells labeled with [U-^13^C_5_]glutamine at 5 and 11 mM glucose concentrations. Data represent means ± SEM, ∗∗∗*p* < 0.01 (n = 3). *B*, absolute exchange flux through isocitrate dehydrogenase (V_IDH Exch_) in *G6pc2* WT and KO βTC3 cells labeled with [U-^13^C_5_]glutamine at 5 and 11 mM glucose concentrations. Data represent means ± SEM, ∗∗∗*p* < 0.01 (n = 3). *C*, oxidative (V_IDH Net_) and reductive fluxes (V_IDH Exch_) relative to total citrate synthase flux (V_CS_) in *G6pc2* WT and KO βTC3 cells labeled with [U-^13^C_5_]glutamine at 5 and 11 mM glucose concentrations. Data represent means ± SEM, ∗∗*p* < 0.05, ∗*p* < 0.10 (n = 3). *D*, cytosolic NADH/NAD^+^ assessed by pyruvate/lactate ratio in *G6pc2* WT and KO βTC3 cells at 5 and 11 mM glucose concentrations. Data represent means ± SEM, ∗∗*p* < 0.05 (n = 3). *E*, cytosolic NADPH/NADP^+^ assessed by malate/pyruvate ratio in *G6pc2* WT and KO βTC3 cells at 5 and 11 mM glucose concentrations. Data represent means ± SEM, ∗∗∗*p* < 0.01, ∗∗*p* < 0.05 (n = 3). *F*, mitochondrial redox state (NAPDH/NADP^+^) indicated by the [α-KG][NH_4_]/[Glu] ratio in *G6pc2* WT and KO βTC3 cells at 5 and 11 mM glucose concentrations. Data represent means ± SEM, ∗∗∗*p* < 0.01 (n = 3). KO, knockout; WT, wild-type.

Our assessment of metabolic fluxes suggested the activation of reductive IDH flux and NADPH-producing pathways that have previously been implicated in augmenting GSIS (13, 14). To further investigate the increase in reductive carboxylation seen in KO cells, we analyzed the MID of intracellular citrate (Fig. 6*A*). Oxidation of labeled glutamine through the forward progression of the CAC results in M + 4 enrichment of citrate. In contrast, M + 5 citrate is indicative of reductive carboxylation of α-KG by IDH2 (13). A significantly lower (M + 4)/(M + 5) ratio was observed in KO cells, consistent with a two-fold increase in IDH exchange flux estimated by ^13^C MFA at both glucose concentrations (Fig. 6*B*). To test if the increased IDH exchange flux is secondary to increased mitochondrial flux, we normalized the metabolic fluxes to CAC activity (V_CS_). Even after this normalization, the reductive IDH flux was significantly higher in KO cells compared with WT cells at both glucose concentrations (Fig. 6*C*). This shows that if an equal amount of carbon enters the CAC of WT or KO cells, the KO cells would route a higher percentage through the reductive carboxylation pathway.

The redox state of β-cells during glucose stimulation is tightly correlated with their capacity for GSIS (13, 15). Therefore, using classical equations and metabolite concentrations (Fig. S8, see Experimental procedures) (16), we determined the metabolic redox state in WT and KO cells. Cytosolic NADH/NAD^+^ was unchanged at 5 mM glucose concentration but was increased at 11 mM glucose concentration in KO cells (Fig. 6*D*). This is consistent with a 55% decrease in pyruvate excretion flux (Fig. S4, *A* and *B*) along with a 60% increase in V_LDH_/V_GK_ flux in KO cells (Fig. 5*A*) from 5 to 11 mM glucose concentration. Additionally, increased malic enzyme and IDH activity was associated with significant elevation in cytosolic NADPH/NADP^+^ ratios at both 5 and 11 mM glucose concentrations (Fig. 6*E*). In agreement with the increase in cytosolic NADPH/NADP^+^ ratio, the mitochondrial NADPH/NADP^+^ fraction was reduced in the KO cells at both glucose concentrations (Fig. 6*F*). These results can be explained by the increase in IDH exchange flux, which has been proposed as a mechanism to transfer reductant (in the form of NADPH) from the mitochondria to the cytosol through the concerted action of IDH1/2 isozymes and the mitochondrial citrate/isocitrate carrier (13). Importantly, flux through the reductive carboxylation and the malic enzyme pathways was upregulated by 67% and 32% in the KO cells, respectively, independent of the glycolytic flux (Fig. 5*C*). Consistent with these flux changes, gene expression analysis revealed substantial upregulation of cytosolic malic enzyme (*Me1*) in KO cells at 5 mM glucose (Fig. S9). Although changes in IDH flux were not strongly coupled to changes in *Idh1/2* expression, the activity of this enzyme is largely controlled posttranscriptionally by acetylation/deacetylation (17). Overall, these results show that ablation of *G6pc2* alters the cytosolic and mitochondrial redox state through upregulation of pathways connected with cytosolic NAD(P)H production, separate from its effects on glycolytic metabolism.

## Discussion

**Figure 7:**
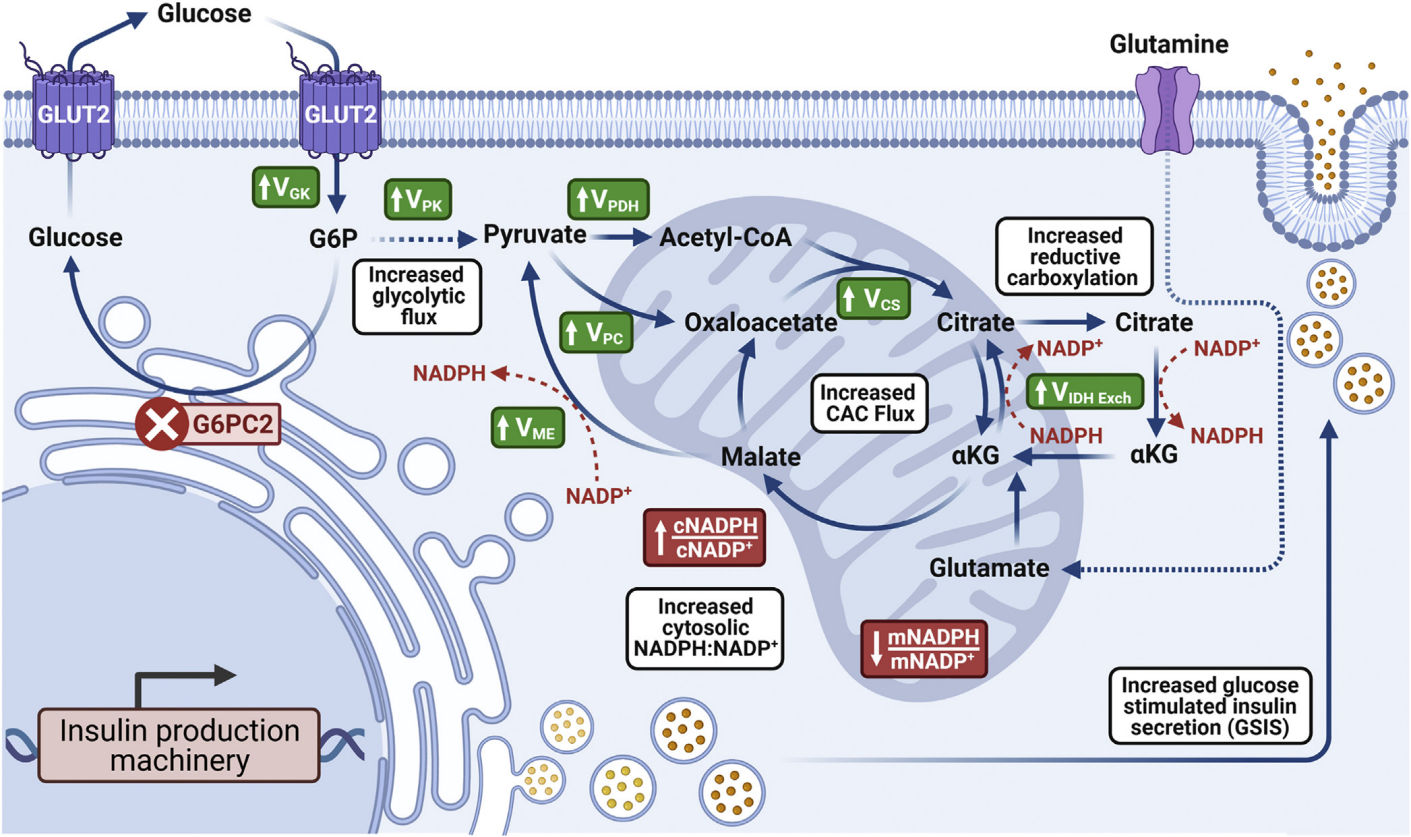
**Schematic illustrating the effect of *G6pc2* KO on oxidative metabolism, energetics, and insulin secretion of βTC3 cells.** Ablation of *G6pc2* leads to increased glycolytic and citric acid cycle (CAC) flux. Cyclic flux through IDH and other putative NADPH-producing pathways is also upregulated along with an increase and a decrease in cytosolic(c) and mitochondrial(m) NADPH:NADP^+^ ratios, respectively. A combination of these metabolic perturbations, previously associated with increased GSIS (14), result in enhanced insulin production and release. Created with BioRender.com. GSIS, glucose-stimulated insulin secretion; IDH, isocitrate dehydrogenase.

Prior studies on G6PC2 have shown that it negatively regulates GSIS and elevates FBG *in vivo* (4). However, while G6PC2 is predicted to control the fate of G6P, its effect on islet metabolism remains largely unknown. Here, using ^13^C flux analysis, we have quantified the metabolic effects of G6PC2 loss on β-cell metabolism and GSIS. Our results show that deletion of *G6pc2* not only affects glycolytic flux but also, as expected, increases CAC flux. However, even after normalizing for the increased glycolytic flux, an increase in CAC flux and NADPH-producing pathways is still apparent (Fig. 7). As observed in *G6pc2* KO islets, we see that the deletion of *G6pc2* in βTC-3 cells increases GSIS. More importantly, we show, by normalizing for the increased glycolytic flux, that while this increase in GSIS is largely fueled by elevated glycolytic flux, it is also bolstered by the influence of G6PC2 on downstream mitochondrial pathways. Additionally, these findings challenge the existing dogma that GK alone regulates glycolytic flux and show that G6PC2 plays a pivotal role in glucose sensitivity of β-cells.

Our results here extend the previous findings on the metabolic control exerted by glucokinase on glycolysis (18, 19). Our revised model here suggests that GK and G6PC2 together regulate glycolytic flux, as the loss of the latter increases glycolytic flux and insulin secretion. Glycolytic flux and insulin secretion in β-cells have also been associated with the PPP, which is an important generator of NADPH and precursor nucleotides (20, 21). Our metabolic model showed minimal oxidative flux through the pentose phosphate pathway in both WT and KO cells, although we did see a significant increase in the ratio of cytosolic NADPH to NADP^+^. Also, our model estimated significant upregulation of pyruvate kinase flux (V_PK_), which recently has been implicated as a dominant regulatory node of glycolysis and insulin secretion (22). The increase seen in V_PK_ in our steady-state metabolic model is secondary to the increase in V_GK Net_, indicative of the latter stage of the two-state model proposed by Lewandowski *et al.* (22). While others have suggested mitochondrial GTP and PEP cycling as insulin amplifying signals (23, 24), we measured negligible enrichment in PEP post incubation with ^13^C_5_-glutamine labeled media (Fig. S10). However, it should be noted that PEP cycle and PEPCK-M have been shown to be active prior to membrane depolarization (22), whereas our model captures oxidative steady-state metabolism after depolarization and calcium entry when PDH and CAC flux is high and PEPCK-M activity is minimized.

As expected, increasing the glucose concentration in the parental, WT and KO βTC3 cells led to an overall increase in nutrient uptake and product excretion fluxes. Several potential pathways have been implicated in regulating excess fuel detoxification in β-cells, including pyruvate, lactate, alanine, glutamate, and more recently, free fatty acids (FFA) and glycerol (25, 26). A newly discovered mammalian glycerol-3-phosphate phosphatase (G3PP) has been associated with linear increases in glycerol production with increasing glucose concentrations within murine pancreatic islets (25, 27). The inclusion of this pathway, along with the enrichment measurements of glycerol-3-phosphate and glycerol, in our metabolic model showed a >2-fold increase in glycerol release flux in KO cells at both glucose concentrations (Figs. 4, *B* and *C* and S4, *A* and *B*). However, compared with other metabolites, the overflow of carbon through G3PP was still limited in magnitude. Instead, we saw that increased oxidative metabolism in KO cells is supported by an increase in the net uptake and excretion of several metabolites centered on the pyruvate node (Fig. S4). At lower glucose concentrations, relative to V_GK net_, only pyruvate release was significantly elevated with a decrease in V_LDH_ and no changes in alanine excretion (Fig. 5*B*). However, at higher glucose concentrations, we saw increased ratios of V_Pyr__R__elease_/V_GK__N__et_, V_LDH_/V_GK__N__et_ and V_ALT_/V_GK__N__et_ in the KO cells (Figs. 5*B* and S4*B*). This is consistent with recent findings (26), wherein pyruvate responds most strongly to increasing fuel pressure in β-cells, potentially to maintain redox equilibrium. Interestingly, the metabolic flux analysis results (Fig. 4, *B* and *C*) also showed a concurrent increase in glutamine uptake flux with rising glucose concentrations, fueling CAC activity along with glycolysis. These results indicate that despite the loss of G6PC2, a tight coupling between glycolysis and mitochondrial metabolism is maintained, a hallmark of physiological β-cell function (28).

While G6PC2 is closely connected with glycolysis, our studies also showed the upregulation of several mitochondrial pathways, independent of increased glucose uptake rate, which are associated with enhancements in insulin secretion. Similar to our results (Fig. 7), increased anaplerosis through pyruvate carboxylase (29), enhanced pyruvate recycling *via* malic enzyme (30), and upregulation of reductive flux through the isocitrate–IDH1/IDH2 pathway (13) have all been associated with upregulation in GSIS. Consistent with increased flux from V_ME_ and V_IDH_, we also measured an increase in cytosolic NAPDH:NADP^+^ ratio along with a slight but significant decrease in mitochondrial NADPH:NADP^+^ ratio. Cytosolic NADPH production has previously been shown to drive the reduction of glutathione (GSH) by GSH reductase (31, 32). This leads to the activation of glutaredoxin (GRX1), which mediates the reduction and activation of sentrin/SUMO-specific protease 1 (SENP1) (31). SENP1 then acts as a deSUMOylase that removes SUMO peptides from secretory granule-trafficking proteins to enhance insulin exocytosis (33). Increased flux through NADPH-producing pathways due to the loss of G6PC2 (Fig. 5*C*) thus may be responsible for increased GSIS in the KO cells (Fig. 3). Additionally, at higher glucose concentrations, we also saw an increase in cytosolic NADH:NAD^+^ ratio along with upregulation of V_LDH_ and V_Lac Release_, factors indicative of hyperglycemia (34). Together these results indicate that reductive CAC flux, an important coupling factor that regulates insulin secretion (13), is increased upon the loss of G6PC2.

While the glucose-6-phosphatase activity of G6PC2 can directly influence the cytosolic concentration of G6P present in the β-cells and hence regulate glycolytic flux, changes in G6P levels can affect ER calcium accumulation through the metabolite’s ability to lower sarco-ER calcium ATPase (SERCA) activity (35). In addition, inhibition of glucose-6-phosphatase has been shown to lower ER Ca^2+^ sequestration in permeabilized islets (36). Therefore, the glycolytic and mitochondrial flux increases seen here (Fig. 5, *A* and *C*) due to the knockout of G6PC2 may be directly associated with changes in calcium signaling in the ER and the cytosol of βTC3 cells. Regulation of mitochondrial pathways by calcium, through allosteric interactions or otherwise, is well understood for many enzymes in central carbon metabolism. Increased calcium levels are known to activate multiple dehydrogenases in the CAC, such as isocitrate dehydrogenase and α-ketoglutarate dehydrogenase, stimulating the respiratory chain and ATP supply (37). More importantly, consistent with our results (Fig. 5*D*), previous studies in *G6pc2* KO mice islets have also shown elevated cytoplasmic calcium levels along with enhanced glycolytic flux (2, 4). Our flux, cytoplasmic calcium, and redox data suggest that G6PC2, potentially through the regulation of calcium signaling, exerts control not only on glycolytic but also mitochondrial metabolism. Future studies will focus on better understanding the impacts of G6PC2 loss on calcium signaling in the ER, mitochondria, and cytosol.

Several genome-wide association studies (GWAS) have identified multiple genetic variants in *G6PC2* that are associated with FBG levels (38, 39), with studies suggesting that single-nucleotide polymorphisms (SNPs) in *G6PC2* affect RNA splicing (40), protein expression (41, 42), and DNA methylation (43). Consistent with previous findings (4), our results here show that knockdown of G6PC2 increases insulin secretion at submaximal glucose concentrations. Also, our study shows an increase in intracellular insulin content upon ablation of *G6pc2*. A different result was seen in *G6pc2* KO islets where no change in intracellular insulin content was observed despite increased insulin secretion (4). These apparently inconsistent results may partly be explained by differences in the duration of glucose stimulation. Unlike Pound *et al.* (4), where the cells were exposed to glucose for 30 min, in our study we incubated the cells in glucose for 24 h. This may have resulted in increased insulin mRNA transcription and translation, since longer incubations have been shown to upregulate proinsulin mRNA levels (44).

Our metabolic model (Fig. 4*A* and Table S1) relies upon fundamental modeling assumptions that introduce some inherent limitations. Firstly, we assume that the citrate enrichment is equivalent to that of isocitrate. This is a safe assumption since no carbon rearrangements are known to occur at the aconitase reaction at equilibrium (45). Secondly, the oxidative IDH reaction modeled in our network does not differentiate between the flux through cytosolic IDH (IDH1) and mitochondrial IDH (IDH2/3) and represents the total flux through both enzymes. Given the identical carbon transitions between IDH isoforms, deconvolution of these pathways is inherently challenging, and future studies requiring the expression of exogenous reporter systems may be needed to resolve compartment-specific IDH fluxes (46). Third, use of only glucose and pyruvate measurements, two metabolites typically used to assess the contribution of PPP to glycolysis (47), provided limited resolution of pentose phosphate pathway flux. Future studies, benefiting from the use of LC-MS/MS platforms, will be needed to measure enrichment in PPP intermediates to better resolve the intermediary fluxes within the pathway. Fourth, while these studies rigorously assessed the metabolic effects of *G6pc2* ablation in βTC3 cells, the development of targeted G6PC2 inhibitors would facilitate further studies of G6PC2 function in a broader range of experimental systems. Future studies will be focused on validating the metabolic effects of such inhibitors, if and when they become commercially available. Finally, although all data pertaining to knockout of *G6pc2* confirms its role as a negative regulator of oxidative metabolism, operation of the pathways described herein remains to be tested in primary mouse and human islets. While rat islets provide a useful model for investigating genetic regulation of FBG and GSIS, as shown previously (11), *G6pc2* is a pseudogene in rats; thus, rat-derived cells are unsuitable for G6PC2 loss-of-function studies. Nevertheless, mouse and human islet studies are feasible but challenging in light of the longer incubation times needed for ^13^C-MFA, leading to *ex vivo* culture adaptions, feedback effects due to media insulin accumulation along with functional heterogeneity of human islet aliquots obtained from different donors.

In conclusion, the data presented in this study show that G6PC2 acts as a negative regulator of oxidative metabolism and GSIS. Our findings suggest that G6PC2, together with GK, regulates glycolysis, citric acid cycle activity, and pathways that control NADPH and insulin production. More importantly, these results validate our hypothesis that knockout of *G6pc2* causes a leftward shift in the dose–response curve for GSIS and is a potential target for enhancing insulin secretion. Since elevated *G6PC2* expression is associated with increased FBG levels and thus heightened risk for cardiovascular-associated mortality (CAM) *in vivo* (40, 48), our study also suggests that G6PC2 inhibitors would be useful for lowering FBG and thereby the risk of CAM. However, one needs to be judicious in interpreting these findings as recent studies suggest that under specific physiological conditions, such as prolonged fasting and ketogenic feeding, G6PC2 offers protection against hypoglycemia (10). Future studies will involve understanding the direct and/or indirect signals that result in the metabolic changes brought about by the deletion of *G6pc2*. Finally, deeper exploration of the potential role of G6PC2 in development of, or compensation for, β-cell failure in T2D remains to be explored in genetic and dietary animal models.

## Experimental procedures

### Cell culture

The βTC3 cell line (49) was cultured in RPMI 1640 medium (Thermo Fisher; Cat no. 11875-093) supplemented with 10% fetal bovine serum (Sigma; Cat. No. F2442) and 1X penicillin-streptomycin (Gibco; Cat. No. 15140122). Rat islet-derived INS-1 832/13 cells (50) were cultured in RPMI medium supplemented with 10% fetal bovine serum, 0.05 mM β-mercaptoethanol, and 1X penicillin-streptomycin. All cells were cultured at 37 °C in a 5% CO_2_ humid atmosphere.

### Generation of G6pc2 knockout (KO) and control βTC3 cells

*G6pc2* KO and control Cas9-expressing βTC3 cells were generated in a multistep process. We first generated a variant βTC3 cell line that stably expresses Cas9. To achieve this, cells were washed twice with Dulbecco’s phosphate-buffered saline (Gibco; Cat. No. 14190144), dissociated with TrypLE Select Enzyme (Gibco; Cat. No. 12563029), and collected as a single-cell suspension. Cell density was measured, and cells were then transduced in a 12-well plate with a lentivirus encoding Edit-R mKate2-tagged Cas9 nuclease at a multiplicity of infection (MOI) of 2.0 under conditions specified by the manufacturer (Dharmacon; Cat. No. VCAS11863). This virus confers constitutive mKate2-tagged Cas9 expression driven by the CMV promoter. By utilizing a 2A self-cleaving peptide, both the fluorescent mKate2 reporter and Cas9 are expressed within the same mRNA strand and translated into two separate proteins. Antibiotic-free complete growth medium (RPMI 1640 (Gibco; Cat. No. 11875093), supplemented with 10% FBS (Sigma; Cat. No. F2442) was added to the transduced cells at a 1:3 volume ratio (transduction media: antibiotic-free complete growth media) after 5 h, and medium was changed every 48 h. Once cells reached a confluency of ∼5 million cells/well (∼6 days), they were dissociated with TrypLE Select Enzyme and resuspended in phenol-red free RPMI medium (Gibco; Cat. No. 11835030) supplemented with 10 mM HEPES buffer (Gibco; Cat. No. 15630106) and antibiotic-antimycotic solution (Gibco; Cat. No. 15240062).

Transduction and Cas9 integration were confirmed under a fluorescence microscope prior to FACS. Cells with high fluorescence signal were clonally sorted onto 96-well plates using a 5-laser FACS Aria III (BD Biosciences) with a 100 μm nozzle. Sorted cells were collected in 150 μl per well antibiotic-containing complete RPMI 1640 growth medium (Gibco; Cat. No. 11875093) supplemented with 10% FBS (Sigma; Cat. No. F2442), 10 mM HEPES (Gibco; Cat. No. 15630080), and 1X penicillin-streptomycin (Gibco, Cat. No. 15140122). Clones were supplemented with an additional 100 μl of antibiotic-containing complete growth medium per well approximately 24 h after sorting. Medium was changed with antibiotic-containing complete growth media every 48 h. mKate2-tagged Cas9-expressing clones were identified using fluorescent image analysis (Leica Microscope Dmi8 and ImageJ). The clones showing the highest fluorescent signal were selected for further analysis and experimental use.

To generate knockouts, *G6pc2* crRNA (Cat. Nos. CM-065306-01, CM-065306-02, CM-065306-03) and synthetic tracrRNA (Cat. No. U-002005-20) were ordered from Dharmacon and resuspended according to the manufacturer’s instructions. Potential off-target sites of Cas9 RNA-guided endonuclease activity were assessed using Cas-OFFinder (51). Results showed zero mismatches or off-target effects of the *G6pc2* crRNA sequences obtained from Dharmacon. Cas9-expressing βTC3 cells (∼10,000 cells per well in four wells of a 24-well plate) were incubated with 25 nM crRNA, 25 nM tracrRNA, and Dharmafect 1 reagent (Cat. No. T-2001-01) in antibiotic-free RPMI medium (Gibco; Cat. No. 11875093) for 48 h at 37 °C. After 48 h, the cells were switched to antibiotic-containing complete growth medium, allowed to recover and then subjected to another round of crRNA:tracrRNA delivery. After three rounds of transfection, the βTC3 cells were expanded and prospectively analyzed for *G6pc2* expression and activity by TIDE (Tracking of Indels by Decomposition) analysis (52) and a stable isotope-based glucose cycling assay (8), respectively. The isolate with the highest KO efficiency and lowest glucose cycling was selected for use in subsequent studies.

### Isotope labeling studies

Prior to labeling experiments, *G6pc2* WT and KO βTC3 cells were washed with PBS and passaged twice in glucose-free RPMI media supplemented with 10% dialyzed FBS and 5 or 11 mM glucose on 10-cm plates. After the second passage, βTC3 cells were grown to ∼60% confluency, washed twice with PBS, and incubated for 24 h in RPMI media supplemented with dialyzed FBS and 2 mM [U-^13^C_5_]glutamine plus unlabeled 5 or 11 mM glucose or with 5 or 11 mM [1,2-^13^C_2_]glucose plus 2 mM unlabeled glutamine. Post incubation, media and cells were harvested and subjected to further analyses as described below.

### Extraction of metabolites and GC-MS analyses

Intracellular metabolites from βTC3 cells were extracted as previously described (53). Briefly, intracellular metabolism was quenched with 2 ml of −80 °C methanol, and cells were scraped into a mixture of 1:1:1 chloroform, methanol, and water. Twenty microliters of 5-mM norvaline and 5-mM [U-^13^C_6_,^2^H_7_]glucose was spiked as an internal standard for metabolite quantification. The aqueous phase was split into two parts, dried, and processed to form either methyloxime *tert*-butyldimethylsilyl (Mox-TBDMS) derivatives of organic and amino acids (54) or the di-isopropylidene propionate (DiO) derivative of glucose (55). Media metabolites were quantified by adding 20 μl of 5-mM norvaline in 50 μl of media followed by cold acetone precipitation. Calibration standards with known amounts of each metabolite were prepared and derivatized simultaneously with the extracted samples for absolute quantification of metabolite abundances. Derivatized samples were analyzed by GC-MS. Sample volumes of 1 μl were injected in a 5:1 split in an Agilent 7890A gas chromatography system equipped with two HP-5 ms (15 m × 0.25 mm × 0.25 μm; Agilent J&W Scientific) capillary columns and interfaced with an Agilent 5977C mass spectrometer. Previously defined temperature programs for Mox-TBDMS (54) and DiO (56) derivatizatives were used for data collection. Derivative peaks were integrated using a custom MATLAB function (8) to obtain mass isotopomer distributions (MIDs) for the metabolite fragment ions shown in Table S2. Measurement uncertainty was assessed by calculating the root-mean-square deviation between the MID of unlabeled standards and the theoretical MID computed from the known abundances of naturally occurring isotopes. Absolute metabolite abundances were normalized to the total number of cells present at the time of sample collection.

### Extracellular uptake and excretion rates

Extracellular uptake and excretion rates of βTC3 cells were determined in triplicate growth experiments. Ten-centimeter tissue culture dishes were seeded at a density of 150,000 cells/ml in 10 ml of RPMI media supplemented with 5 or 11 mM glucose. After incubating parallel dishes for 12 h, 24 h, 48 h, or 72 h, extracellular media was collected and stored at −80 °C for further analysis while the cells were washed with PBS, detached using 0.05% Trypsin-EDTA (Gibco; Cat no. 25300062) and counted using a Cedex XS cell counter (Roche; Cat no. 702070001). Concentrations of media metabolites were then analyzed using GC-MS (see Extraction of metabolites and GC-MS analyses). We integrated the media metabolite concentrations and cell counts at each timepoint into our previously developed MATLAB-based software package Extracellular Time-Course Analysis (ETA) to quantify cell-specific uptake and excretion rates of measured metabolites (57). The spontaneous degradation of glutamine to ammonia and pyrrolidone carboxylic acid was included in the specific rate calculations, and the degradation rate was determined to be 0.0031 h^−1^ by measuring glutamine disappearance in control cell-free plates. Evaporation rates determined in these control plates were found to be negligible in comparison to cell-specific metabolic rates.

### ^13^C metabolic flux analysis

^13^C MFA was performed by minimizing the sum-of-squared residuals (SSR) between model-simulated and experimentally determined measurements. The Isotopomer Network Compartmental Analysis (INCA) software package (58, 59) was used to develop a model of β-cell metabolism (Table S1) and estimate fluxes by fitting the model to the experimental datasets. Extracellular uptake rates and metabolite enrichment measurements were provided as inputs into the INCA model for flux analysis of βTC3 cell cultures. The standard error in these measurements was set to either the root-mean-square deviation of the unenriched control samples or the standard error of the mean (SEM) obtained from biological replicates, whichever was greater. Best-fit metabolic flux solutions were determined for each experiment by least-squares regression of the experimental measurements to the isotopomer network model. To ensure that a global solution was obtained, flux estimations were repeated a minimum of 25 times from randomized initial guesses. A Chi-square test was used to assess goodness-of-fit, and a sensitivity analysis was performed to determine 95% confidence intervals associated with the calculated flux values. Although the metabolic model does not directly capture the flux through G6PC2 (V_G6PC2_), the combination of ^13^C metabolite enrichments and net glucose uptake measurements enable estimation of the net flux through glucokinase (V_GK Net_) where V_GK Net_ = V_GK_ – V_G6PC2_.

### Intracellular and media insulin quantification

Cell culture media (∼500 μl) was collected from 10-cm dishes at 12-h intervals (n = 3 per timepoint) for insulin quantification. A previously described (60) islet insulin extraction protocol was adapted to measure insulin content within βTC3 cells. Approximately 5 million βTC3 WT and KO cells were washed four times with ice-cold PBS, prior to 48-h refrigeration in 300 μl of acid alcohol (1 ml concentrated HCl; 110 ml 95% ethanol). After incubation for 48 h, the extract was centrifuged for 10 min at 2000 RPM at 4 °C. Lastly, 200 μl of supernatant was collected and stored at −20 °C for insulin analysis. Insulin from cell extracts and media was quantified using radioimmunoassay (Millipore) by the Vanderbilt Diabetes Research and Training Center Hormone Assay Core.

### Intracellular metabolite quantification and assessment of redox markers

Intracellular metabolites from 8 to 10 million βTC3 WT and KO cells were extracted as defined above (see Extraction of metabolites and GC-MS analyses). Absolute quantification of metabolite amount was performed by running calibration standards along with extracted samples. Cell counts in biological replicates were assessed after detaching and counting them using 0.05% Trypsin-EDTA (Gibco; Cat no. 25300062) and a Cedex XS cell counter (Roche; Cat no. 702070001), respectively. Metabolite amount was normalized to total volume estimated using cell counts and assuming an average volume of a β-cell of approximately 1100 μm^3^ from previous studies (61). For analysis of ammonium, 300 μl sample was diluted by dH_2_O to 1.5 ml and then analyzed using an Ammonia Gas Sensing Electrode (Cat. No. 9512BNWP, Thermo Fisher Scientific) according to the manual. Cytosolic and mitochondrial redox state was estimated using enzymatic equilibrium relations described elsewhere (16). The cytosolic NADH/NAD^+^ was estimated from lactate dehydrogenase equilibrium (*i.e.*, cNADH/NAD^+^ = [Lactate]/[Pyruvate] × 1/K_LDH_; where K_LDH_ = 1.11 × 10^−4^). Similarly, cytosolic NADPH/NADP^+^ was estimated from malate dehydrogenase equilibrium (cNADPH/NADP^+^ = [Malate]/[Pyruvate][CO_2_] × K_MDH_; where K_MDH_ = 34.4 × 10^3^ μM). Lastly, mitochondrial NADPH/NADP^+^ was estimated from glutamate dehydrogenase equilibrium (mNADPH/NADP^+^ = [Glutamate]/[α-ketoglutarate][NH_4_^+^] × K_GDH_; where K_GDH_ = 2.49 × 10^−3^ mM) (16).

### Glucose cycling

Glucose cycling in βTC3 and INS1-832/13 cells was measured using our previously defined approach (8). Briefly, 8 to 10 million cells were incubated for 24 or 72 h at 37 °C in RPMI 1640 medium containing 5 mM or 11 mM glucose in 10-cm dishes. Cells were incubated in either naturally labeled glucose or [1,2,3,4,5,6,6-^2^H_7_]glucose (Cambridge Isotope Laboratories; Cat no. DLM-2062). Following the 24- or 72-h incubation, the supernatant was collected for glucose derivatization and GC-MS analysis (see Extraction of metabolites and GC-MS analyses). The glucose MID was quantified, and absolute glucose concentration was determined through comparison to a standard curve. Glucose cycling and glucose uptake rates were calculated as described previously (8).

### Measurement of cytoplasmic calcium

βTC3 WT and KO cells were cultured in RPMI with 5 mM glucose or 11 mM glucose for 24 h at 37 °C, 5% CO_2_ in 35-mm tissue culture dishes. Cells were loaded with 2 μM Fura-2 AM (InvitrogenTM) for 25 min. Cells were then washed, incubated (for 15 min), and perfused with Krebs-Ringer HEPES buffer (KRHB) containing (mM) 119.0 NaCl, 4.7 KCl, 2.5 CaCl_2_, 1.2 MgSO_4_, 1.2 KH_2_PO_4_, and 10.0 HEPES (pH 7.35 adjusted by NaOH) supplemented with either 5 mM glucose or 11 mM glucose. Baseline Ca^2+^ level was recorded at glucose concentrations indicated in the figure legends. βTC3 K_ATP_ channels were subsequently inhibited with 100 μM tolbutamide in KRHB; only cells that showed functional K_ATP_ channels as determined by tolbutamide-induced Ca^2+^ influx were analyzed. Fura-2 AM Ca^2+^ fluorescence (excited at 488 nm) was measured at 340 nm and 380 nm (F_340_/F_380_) every 5 s as an indicator of intracellular Ca^2+^ using a Ti2 microscope (Nikon) and a back-illuminated sCMOS Prime 95B camera (Teledyne Photometrics).

### Microsome isolation and measurement of glucose-6-phosphatase (G6Pase) activity *in vitro*

Microsome isolation and *in vitro* G6Pase activity measurements were performed as previously described (42). Briefly, βTC3 WT or KO cells in six 3.5-cm diameter dishes were harvested and resuspended in 1 ml of solution containing 50 mM Tris HCl (pH 8.0), 50 mM NaCl, 0.5 mM EDTA, and 10% glycerol at 4 °C. Cells were then sonicated for 30 s (3-s pulses with 10-s pauses). Cell debris was removed by centrifuging the homogenate at 7800*g* for 6 min at 4 °C. The supernatant was then diluted approximately three-fold with the same buffer before being subjected to a high-speed spin (214,000*g*) for 30 min at 4 °C using a Beckman TLA 100.3 rotor to isolate a microsomal membrane fraction, which was then resuspended using 300 to 400 μl of the same buffer.

For the measurement of G6Pase activity, microsomal membranes (75 μl) isolated from WT or KO cells were mixed with 2X reaction buffer (75 μl; 58 mM MES, 42 mM Tris, 100 mM NaCl pH 6.6) or the same 2X reaction buffer supplemented with 4 mM G6P. Reactions were incubated in a 30 °C water bath for 120 min and then placed on ice for 1 min. Reactions were then quenched using a 12% SDS solution (150 μl) and vortexed. A 1:1 mixture of cold P_i_ chelating solution (300 μl; 2% ammonium molybdate: 12% ascorbic acid) was added to each reaction before incubation at room temperature for 5 min. A developing solution (450 μl; 80 mM sodium citrate, 150 mM sodium meta-arsenite, 2% glacial acetic acid) was then added to the reaction and incubated at room temperature for 20 min. The absorbance of the solution at 850 nm was then measured using a plate reader. Comparison of the absorbance value to that of a phosphate standard curve was used to determine G6Pase activity.

### Gene expression analysis

RNA was isolated from ∼2 million βTC3 cells using QIAshredder (Qiagen, Cat no. 79656) and RNeasy Mini Kit (Qiagen, Cat no. 74104), according to manufacturer protocols. To perform quantitative real-time PCR analysis, cDNA was synthesized using the iScript cDNA synthesis kit (Bio-Rad, Cat no. 1708891). Next, this cDNA was diluted tenfold with DI water, mixed with custom designed primer sequences (Integrated DNA Technologies) and iQ SYBR Green Supermix (Bio-Rad), and analyzed on a CFX96 Real-Time PCR System (Bio-Rad). Gene expression was normalized to *Ppia* and WT controls using the 2^−ΔCt^ and 2^−ΔΔCt^ methods, respectively (62). Previously defined primer sequences were used for *G6pc2*, *Hk1*, *Hk4 (Gck)*, *Glut1* (*Slc2a1*), and *Glut2 (Slc2a2)* (2). Other primer sequences used were as follows: *Idh1*, forward 5′- ATGCAAGGAGATGAAATGACACG -3′, reverse 5′- GCATCACGATTCTCTATGCCTAA -3′, *Idh2*, forward 5′- GGAGAAGCCGGTAGTGGAGAT -3′, reverse 5′- GGTCTGGTCACGGTTTGGAA -3′, *Me1*, forward 5′- GTCGTGCATCTCTCACAGAAG -3′, reverse 5′- TGAGGGCAGTTGGTTTTATCTTT -3′, *Me2*, forward 5′- GGCTAAGAGCTGTTACCACTCC -3′, reverse 5′- CGTAAACGCCATTCCCTTGTT -3′.

### Statistical analyses

Unless otherwise specified, data are presented as means ± SEM. Differences between groups were tested using an unpaired parametric *t* test without assuming consistent standard deviations (Welch’s *t* test). Unless otherwise stated in figure legends, significant differences were defined as follows: ∗∗∗*p* < 0.01, ∗∗*p* < 0.05, and ∗*p* < 0.1.

## Data availability

All data described are contained within the manuscript

## Supporting information

This article contains supporting information..

## Conflict of interest

The authors declare no conflict of interest with the contents of this article.
