# Supplementary material for: Glucose-6-phosphatase catalytic subunit 2 negatively regulates glucose oxidation and insulin secretion in pancreatic β-cells
Source: J Biol Chem. 2022 Feb 15;298(4):101729. doi: 10.1016/j.jbc.2022.101729 (PMC8941207; doi:10.1016/j.jbc.2022.101729)
Supplement: Supplemental Figures S1–S10, Tables S1 and S2 [file mmc1.pdf]

Supporting information accompanying research article titled

Glucose-6-phosphatase catalytic subunit 2 negatively regulates glucose oxidation and insulin secretion in pancreatic β-cells

Mohsin Rahim[1], Arya Y. Nakhe[2], Deveena R. Banerjee[2], Emily M. Overway[2], Karin J. Bosma[2], Jonah C. Rosch[1], James K. Oeser[2], Bo Wang[1], Ethan S. Lippmann[1,3,4], David A. Jacobson[2], Richard M. O'Brien[2], Jamey D. Young[1,2] §

[1]Department of Chemical and Biomolecular Engineering, [2]Department of Molecular Physiology and Biophysics, [3]Department of Biomedical Engineering, [4]Department of Neurology, Vanderbilt University, Nashville, TN USA

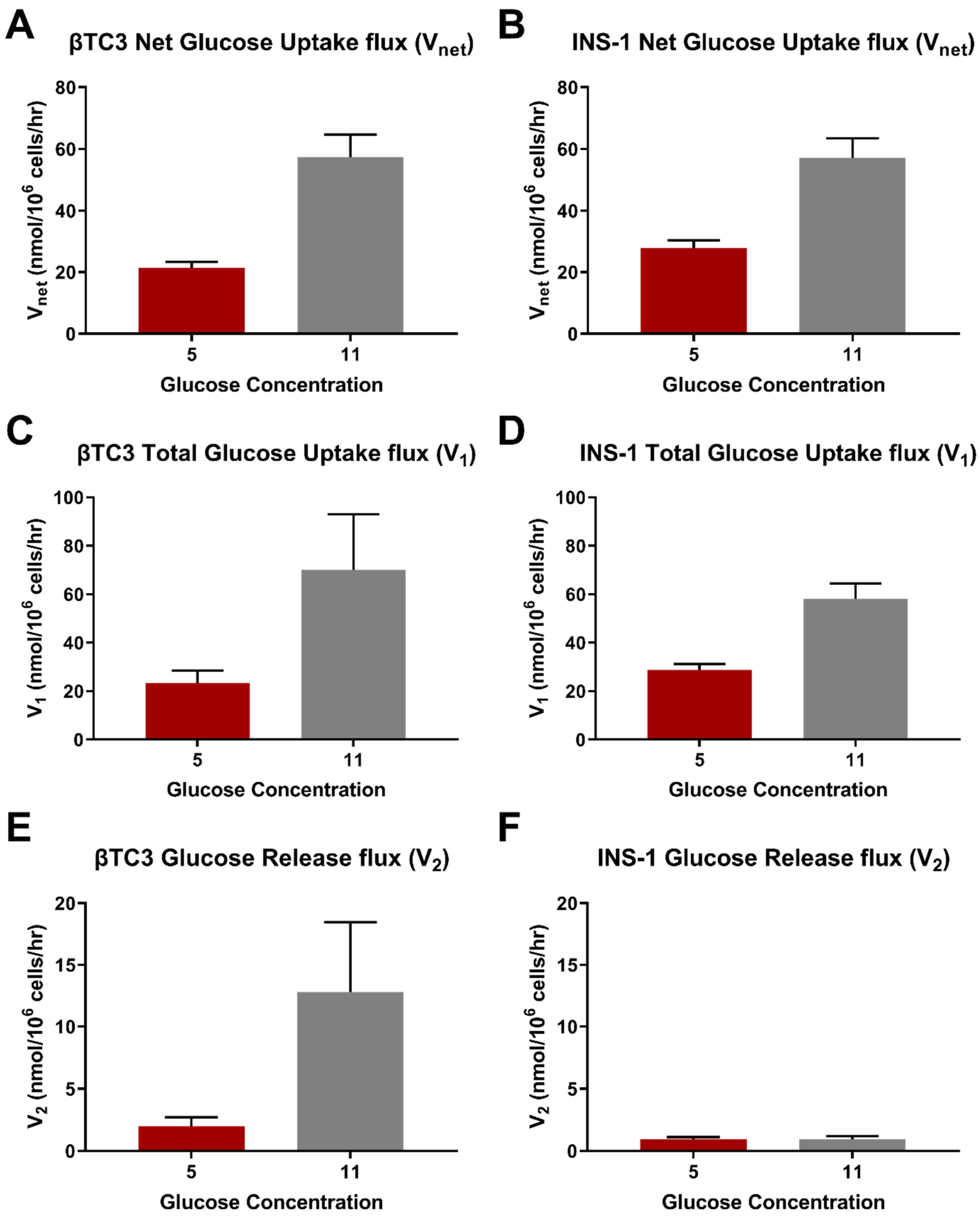

Figure S1: Measurement of glucose uptake and release fluxes in βTC3 and INS-1 832/13 rat insulinoma cells

Measurement of **(A-B)** net glucose uptake flux (vnet), **(C-D)** total glucose uptake flux (v1), and **(E-F)** total glucose release flux (v2) at 5 and 11 mM glucose concentrations in βTC3 and INS-1 832/13 cells. Data represent means±SEM (n=3).

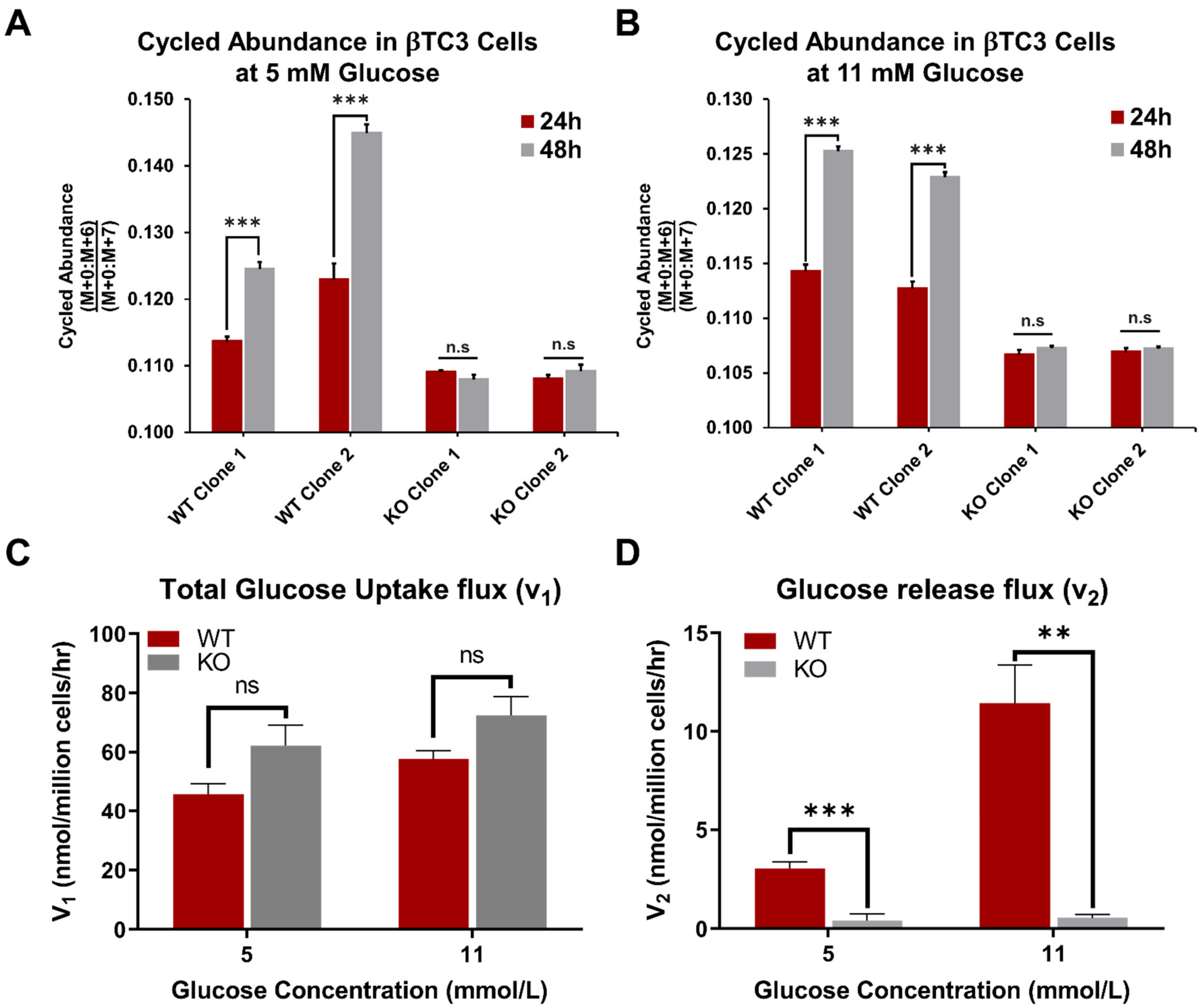

Figure S2: Cycled abundance in two biological isolates of *G6pc2* WT and KO βTC3 single cell clones at 5 and 11 mM glucose concentrations

Cycled abundance, which is the ratio M+0:M+6 isotopomers to M+0:M+7 isotopomers, measured in cell media taken at 24h and 48h after incubation with [1,2,3,4,5,6,6-$^2H_7$]glucose at **(A)** 5 mM and **(B)** 11 mM glucose concentrations. Data represent means±SEM, ****p*<0.01 (n=3). **(C)** Total glucose uptake flux (v_1) and **(D)** Total glucose release flux (v_2) measured in βTC3 *G6pc2* WT and KO cells. Data represent means±SEM (n=3).

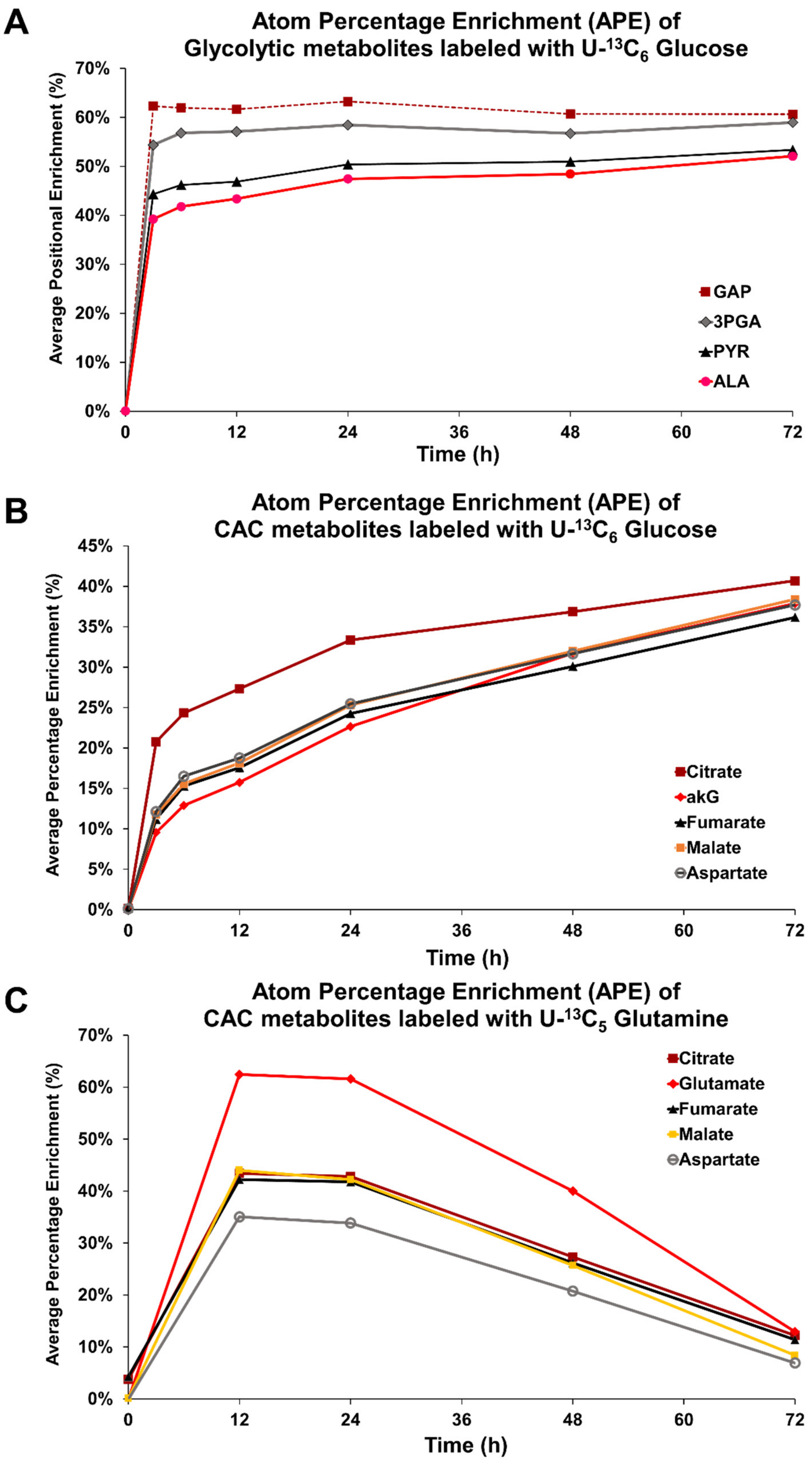

Figure S3: Atom percentage enrichment (APE) in metabolites over time to determine isotopic steady state in isotope labelling experiments

A) APE in glycolytic metabolites reaches steady state within 24h when labelled with 11 mM [U-$^{13}C_6$]glucose.

B) APE in CAC metabolites continues to increase in enrichment over 72h when labelled with 11 mM [U-$^{13}C_6$]glucose.

C) APE in CAC metabolites reaches steady state between 12 to 24h when labelled with 2 mM [U-$^{13}C_5$]glutamine.

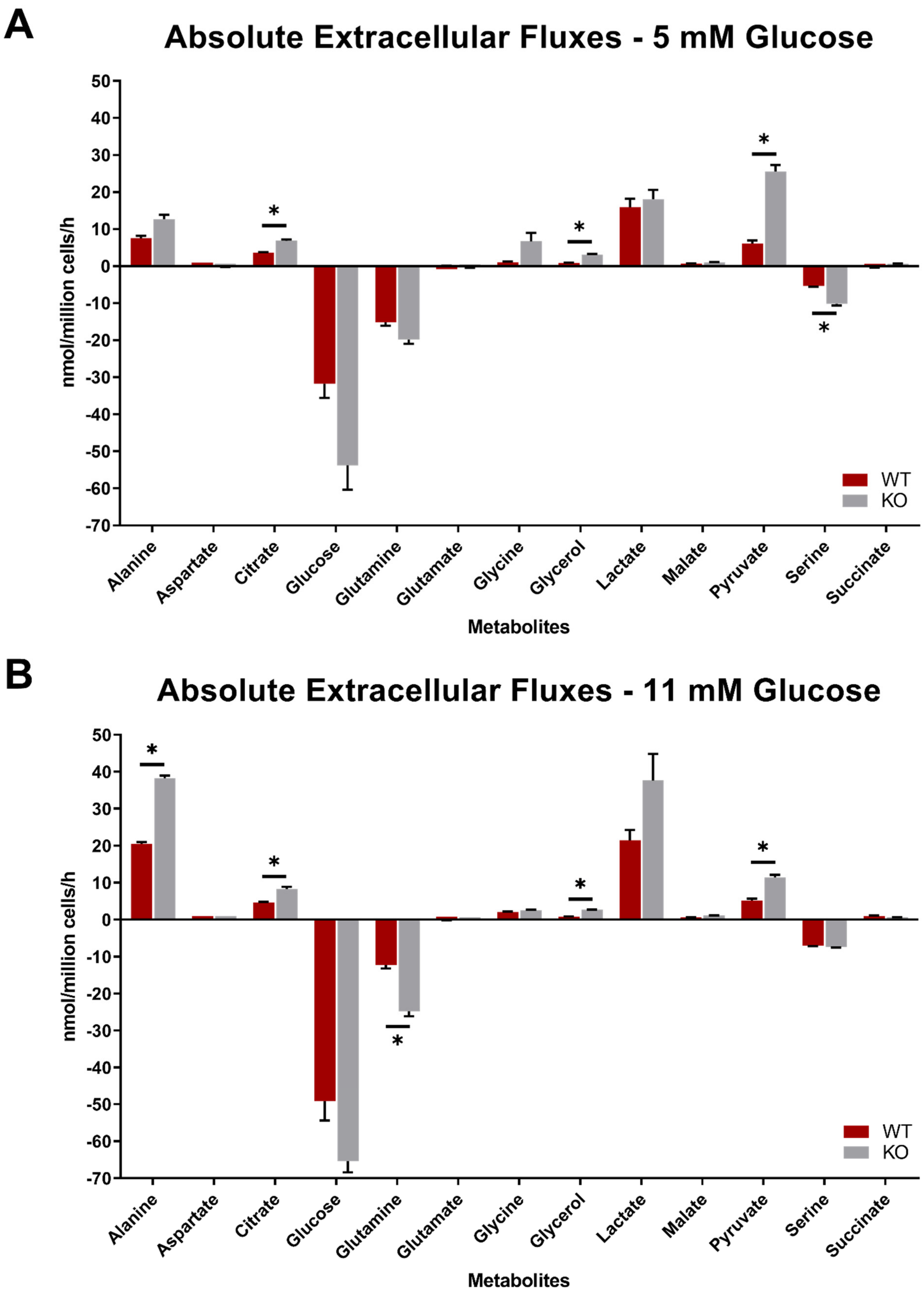

Figure S4: Extracellular uptake and excretion rates in *G6pc2* WT and KO βTC3 cells
Extracellular uptake and excretion measured in *G6pc2* WT and KO βTC3 cells incubated at **(A)** 5 and **(B)** 11 mM glucose concentrations. Positive values represent excretion fluxes while negative values indicate net uptake of metabolite. Data represent means±SEM, *p<0.05 (n=3).

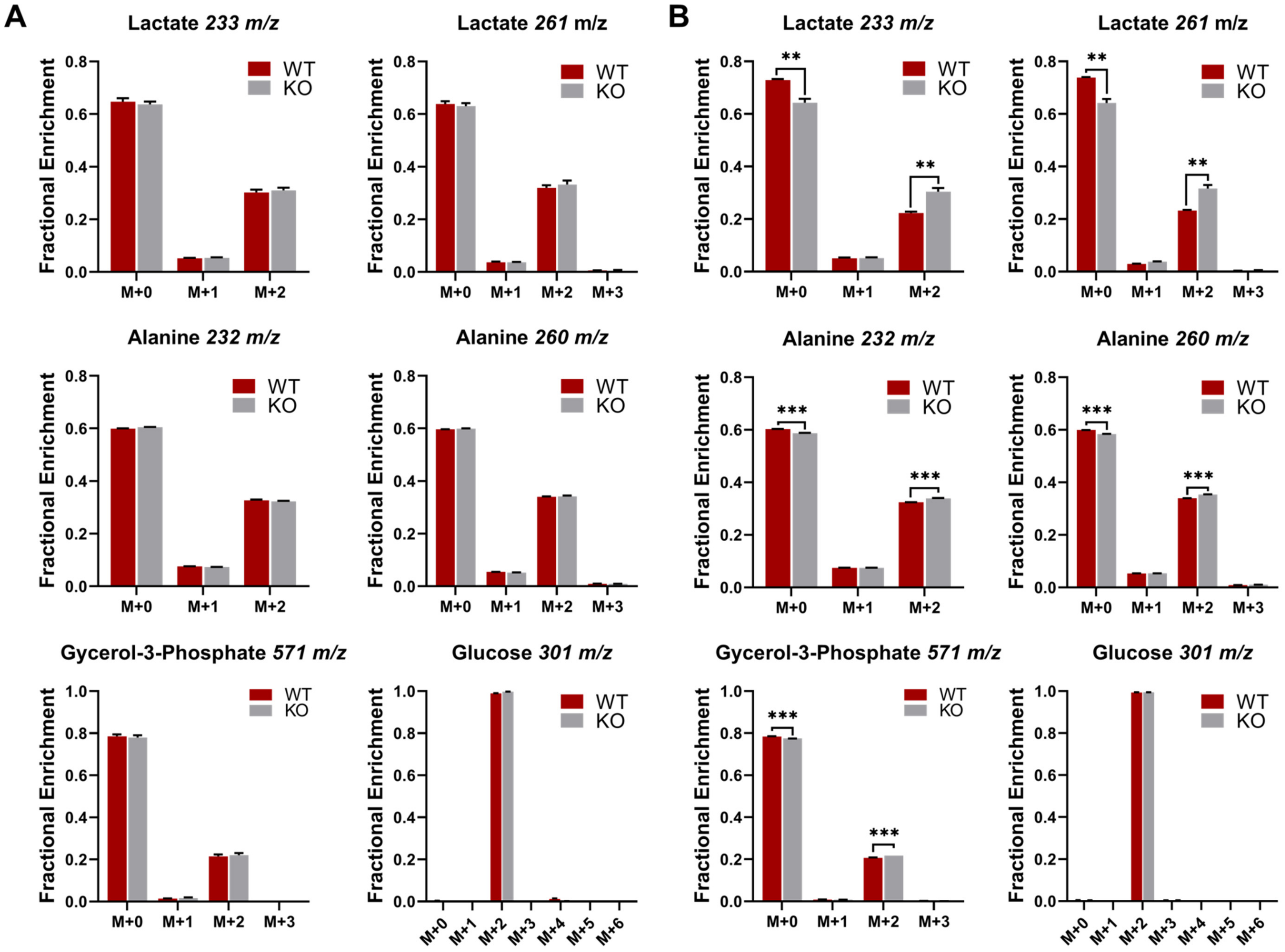

Figure S5: Enrichment of glycolytic metabolites in *G6pc2* WT and KO βTC3 cells labeled with [1,2-$^{13}C_2$]glucose
Intracellular enrichment in glycolytic metabolites measured in *G6pc2* WT and KO βTC3 cells incubated with **(A)** 5 and **(B)** 11 mM [1,2-$^{13}C_2$]glucose. Data represent means±SEM, ***p<0.01, **p<0.05 (n=3).

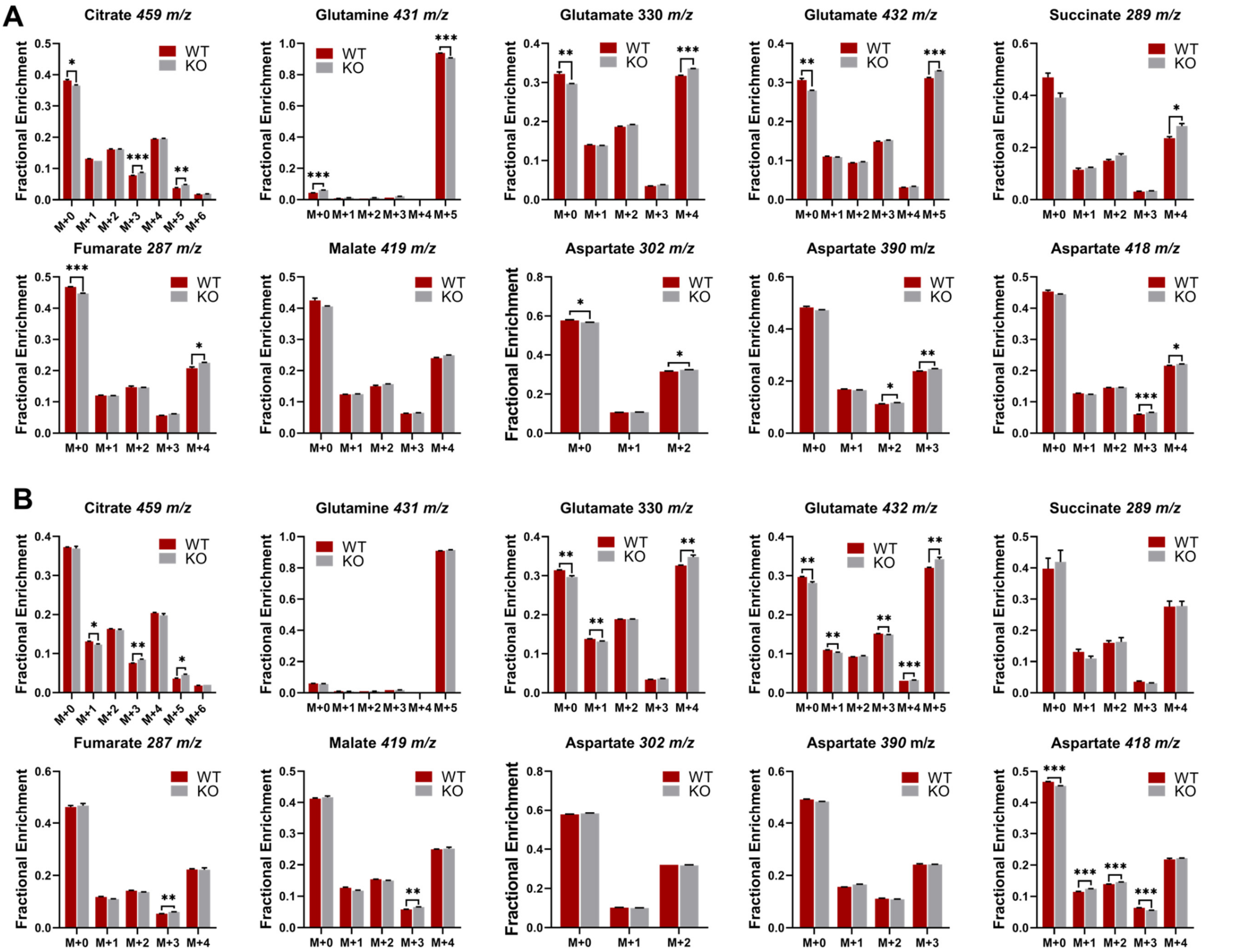

Figure S6: Enrichment of CAC metabolites in *G6pc2* WT and KO βTC3 cells labeled with 2 mM [U-$^{13}C_5$]glutamine

Intracellular enrichment in CAC metabolites measured in *G6pc2* WT and KO βTC3 cells incubated with **(A)** 5 and **(B)** 11 mM glucose. Data represent means±SEM, ***p<0.01, **p<0.05, *p<0.01 (n=3).

A

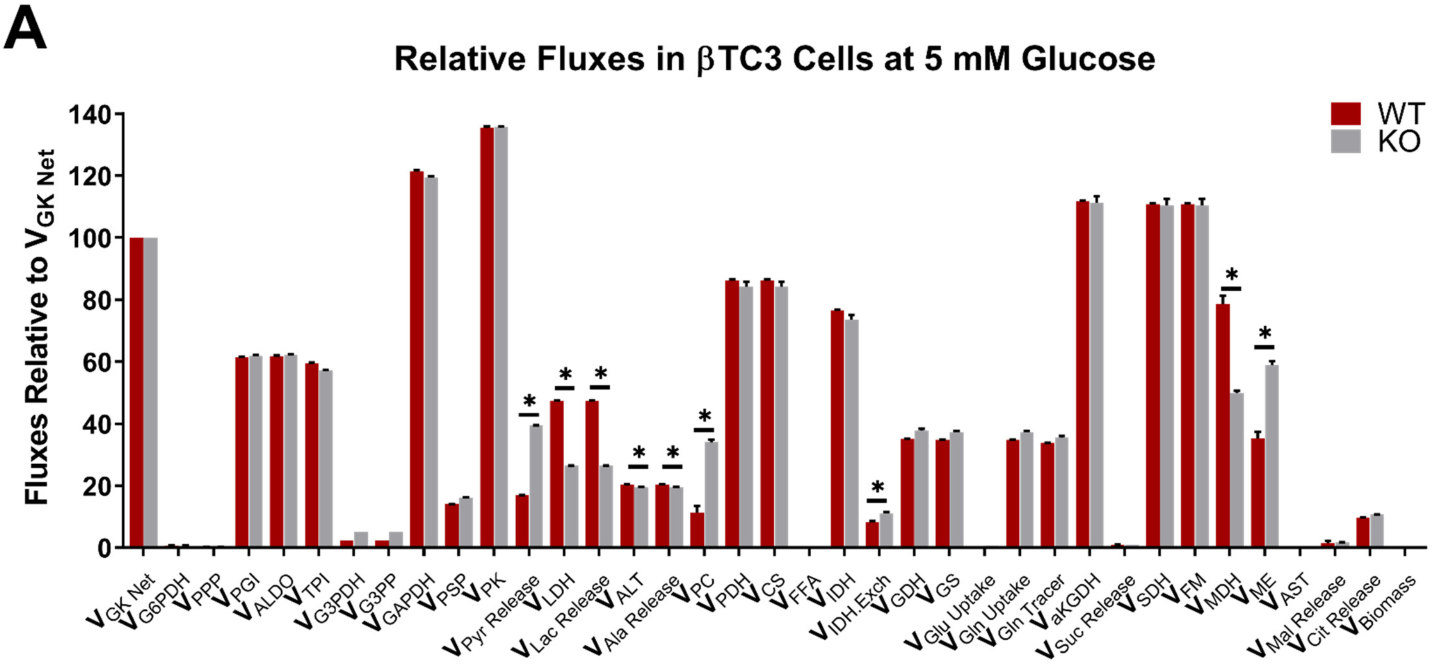

B

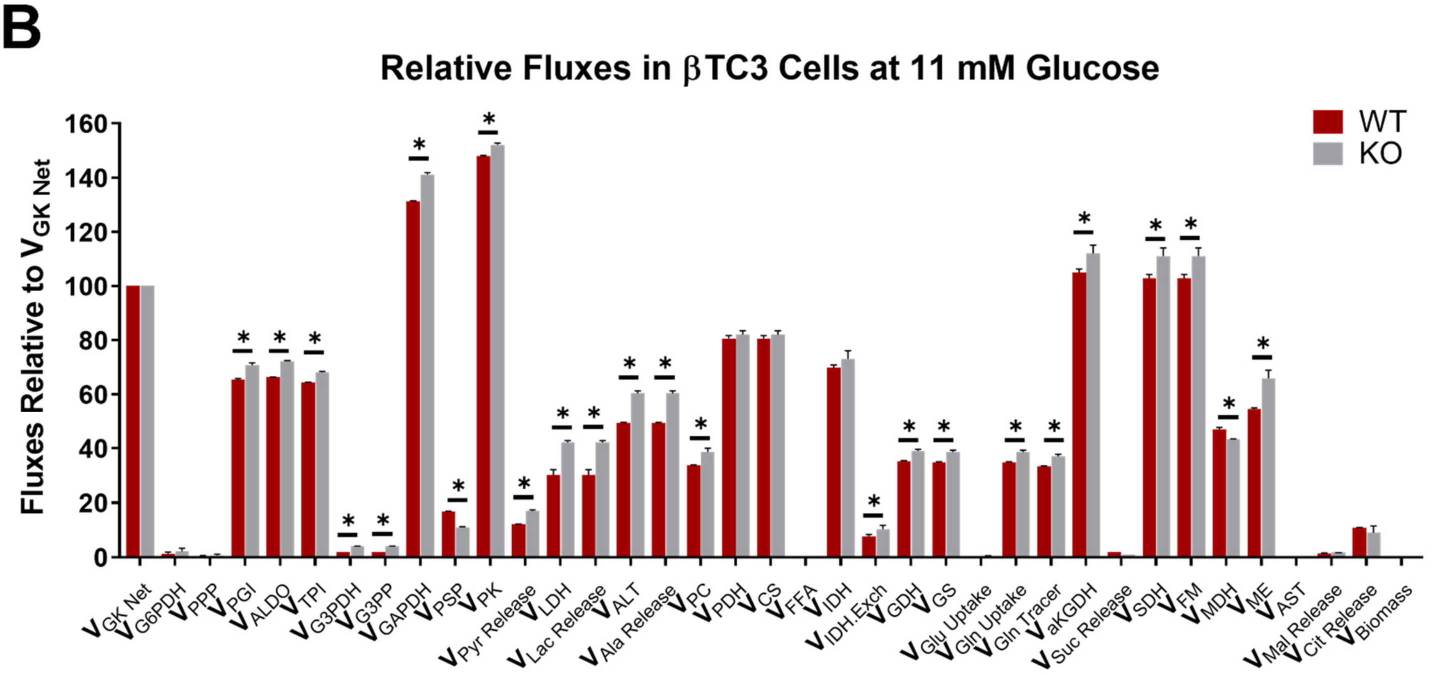

Figure S7: Metabolic fluxes relative to net glucose uptake in *G6pc2* WT and KO βTC3 cells
Metabolic fluxes relative to net glucose uptake ($V_{GK\ Net}$) in *G6pc2* WT and KO βTC3 cells at **(A)** 5 mM and **(B)** 11 mM (right) glucose concentrations estimated using MFA. Data represent means±SEM, *p<0.05 (n=3)

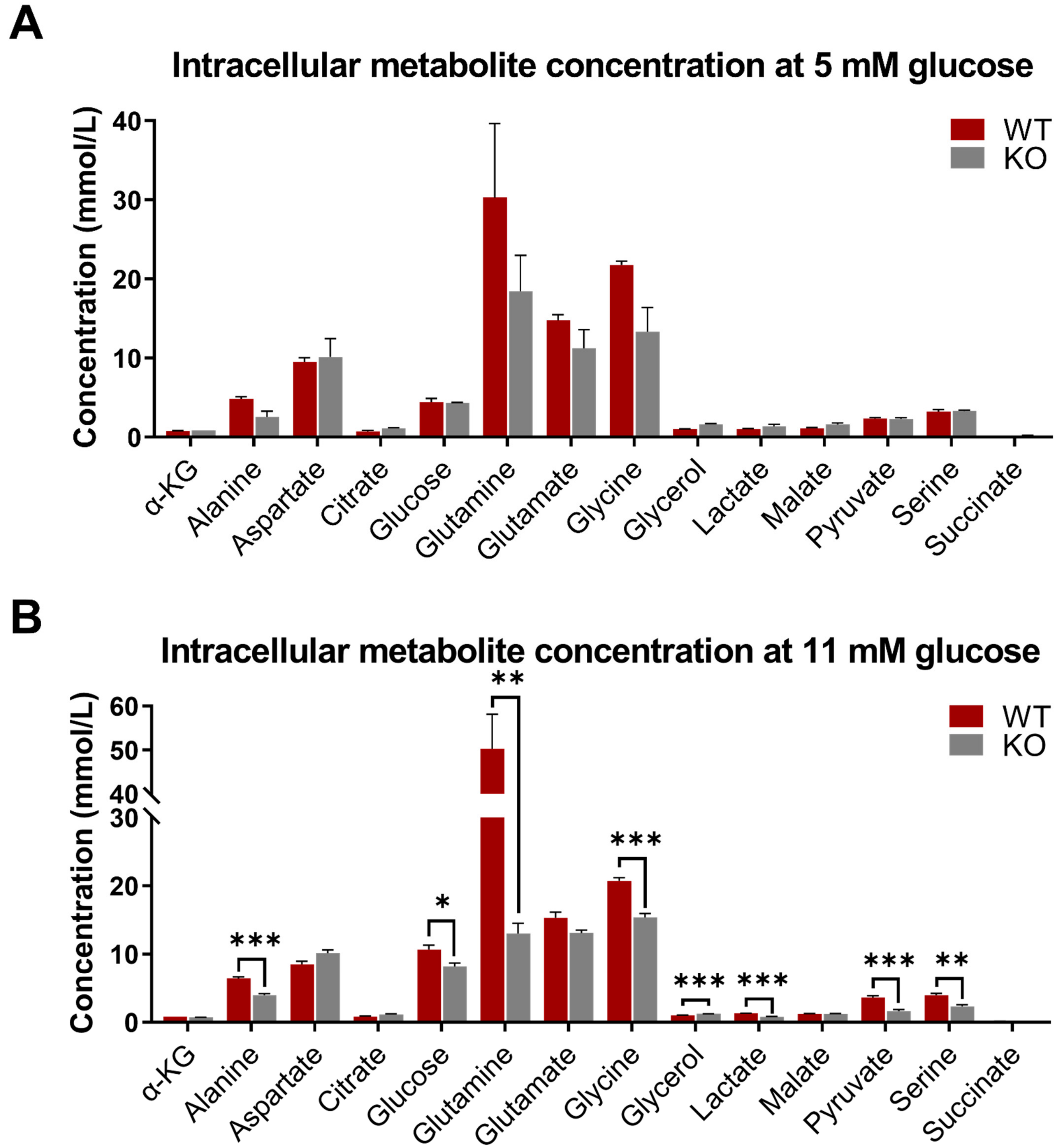

Figure S8: Intracellular metabolite abundance in *G6pc2* WT and KO βTC3 cells labeled with 2 mM [U-$^{13}C_5$]glutamine

Intracellular metabolite abundance measured in *G6pc2* WT and KO βTC3 cells incubated with **(A)** 5 and **(B)** 11 mM glucose. Data represent means±SEM, ****p*<0.01, ***p*<0.05, **p*<0.10 (n=3).

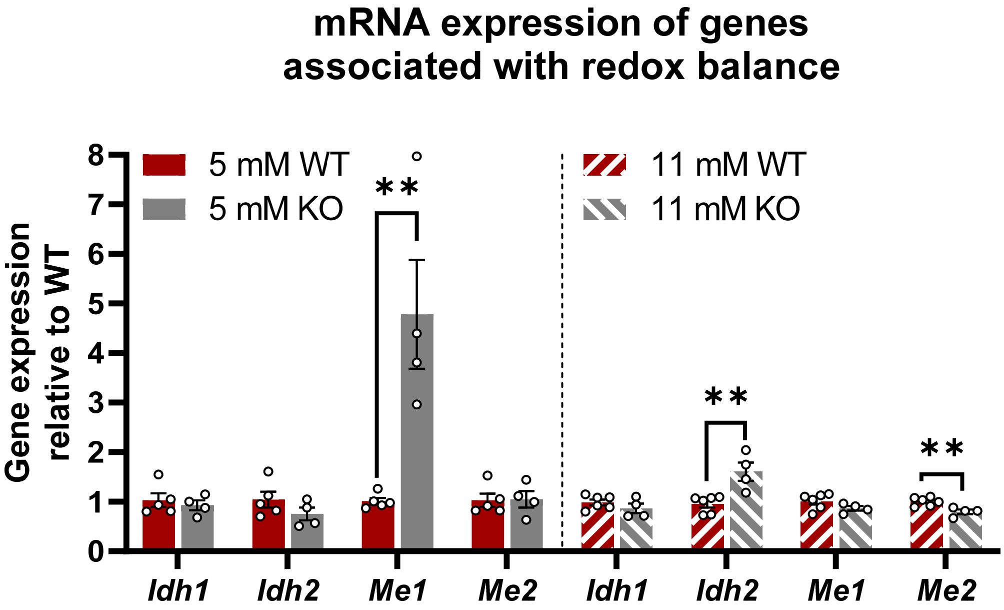

Figure S9: Effects of the loss of *G6pc2* on *expression of* genes regulating redox control. mRNA expression of genes regulating redox metabolism. Data represent means±SEM (n=*3)* relative to expression of the WT, **p<0.05 (n=3).

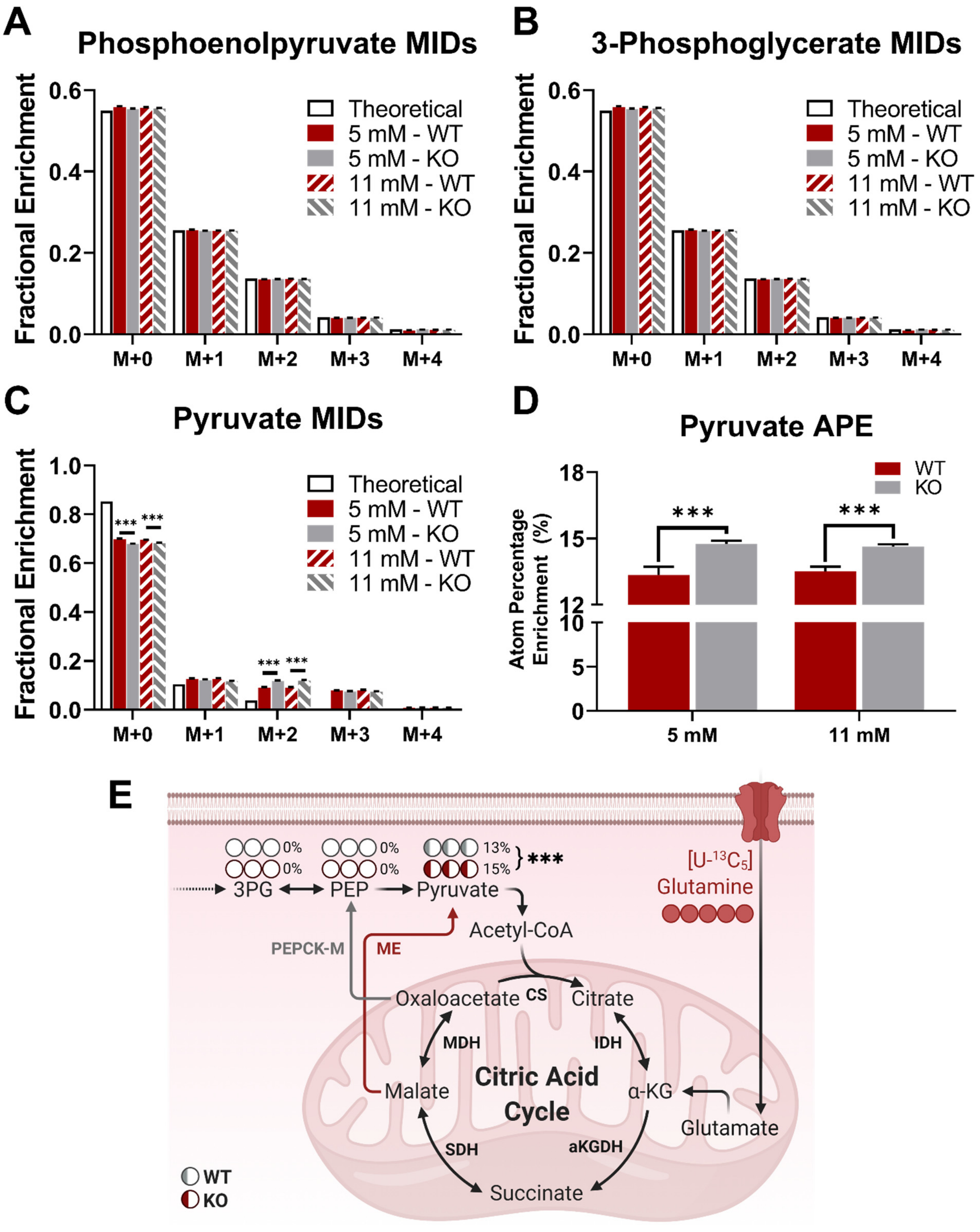

Figure S10: Enrichment patterns in glycolytic metabolites after incubation with 2 mM [U-$^{13}C_5$]glutamine Intracellular enrichment in **(A)** phosphoenolpyruvate (PEP) **(B)** 3-phosphoglycerate (3PG), and **(C)** pyruvate, uncorrected for background abundance in *G6pc2* WT and KO βTC3 cells at 5 and 11 mM glucose concentrations incubated with 2 mM [U-$^{13}C_5$]glutamine for 24h. The theoretical values represent the unlabeled MIDs for each metabolite. **(D)** APE of pyruvate at 5 and 11 mM glucose concentrations **(E)** Schematic showing pathways that can enrich 3PG, PEP and pyruvate when [U-$^{13}C_5$]glutamine is used as tracer. Only pyruvate showed isotopic enrichment while PEP and 3PG had negligible ^{13}C incorporation, suggesting that PEPCK-M is inactive at steady state in βTC3 cells. Data represent means±SEM, ***p<0.01 (n=3).

Table S1. Pancreatic β-cell metabolic reaction network for ^{13}C MFA. (Related to Fig. 4-6 and Table S2) Network maps of β-cell metabolism track carbon atoms through model reactions. Metabolites used to regress fluxes in both compartments are shown in Table S2. Unenriched sources and sinks and "CO_2" are annotated as ".source" and ".sink", respectively. ^{13}C isotopes are introduced into model reactions as ".tracer". Extracellular metabolites are designated as ".ext".

**Glycolysis**	
$V_{Gluc.source}$	Gluc.source (ABCDEF) ⟶ Gluc (ABCDEF)
V_{GK}	Gluc (ABCDEF) ⟶ G6P (ABCDEF)
V_{PGI}	G6P (ABCDEF) ⟶ F6P (ABCDEF)
V_{ALDO}	F6P (ABCDEF) ⟶ DHAP (CBA) + GAP (DEF)
V_{TPI}	DHAP (ABC) ⟷ GAP (ABC)
V_{G3PDH}	DHAP (ABC) ⟶ G3P (ABC)
V_{G3PP}	G3P (ABC) ⟶ Glycerol.ext (ABC)
V_{GAPDH}	GAP (ABC) ⟶ 3PG (ABC)
V_{PSP}	Ser (ABC) ⟶ 3PG (ABC)
V_{PK}	3PG (ABC) ⟶ Pyr (ABC)
$V_{Pyr.exch}$	Pyr (ABC) ⟷ Pyr.ext (ABC)
$V_{Pyr.release}$	Pyr.ext (ABC) ⟶ Pyr.sink (ABC)
V_{LDH}	Pyr (ABC) ⟷ Lac (ABC)
$V_{Lac.exch}$	Lac (ABC) ⟷ Lac.ext (ABC)
$V_{Lac.release}$	Lac.ext (ABC) ⟶ Lac.sink (ABC)
V_{ALT}	Pyr (ABC) ⟷ Ala (ABC)
$V_{Ala.exch}$	Ala (ABC) ⟷ Ala.ext (ABC)
$V_{Ala.release}$	Ala.ext (ABC) → Ala.sink (ABC)
V_{PC}	Pyr (ABC) + CO2 (D) ⟶ Oac (ABCD)
V_{PDH}	Pyr (ABC) ⟶ AcCoA (BC) + CO2 (A)
**Pentose Phosphate Pathway**	
V_{G6PDH}	G6P (ABCDEF) ⟶ P5P (BCDEF) + CO2 (A)
V_{TK1}	S7P (ABCDEFG) + GAP (HIJ) ⟷ F6P (ABCHIJ) + E4P (DEFG)
V_{PPP}	P5P (ABCDE) + P5P (FGHIJ) ⟷ S7P (ABFGHIJ) + GAP (CDE)
V_{TK2}	P5P (ABCDE) + E4P (FGHI) ⟷ F6P (ABFGHI) + GAP (CDE)
**Citric Acid Cycle**	
V_{CS}	Oac (ABCD) + AcCoA (EF) ⟶ Cit (DCBFEA)
$V_{Fat.entry}$	FA (AB) ⟶ AcCoA (AB)
V_{IDH}	Cit (ABCDEF) ⟷ α-kg (ABCDE) + CO2 (F)
V_{GDH}	Glu (ABCDE) ⟷ α-kg (ABCDE)

V_{GS}	Gln (ABCDE) ↔ Glu (ABCDE)
$V_{Glu.entry}$	Glu.ext (ABCDE) ↔ Glu (ABCDE)
$V_{Glu.source}$	Glu.source (ABCDE) ⟶ Glu.ext (ABCDE)
$V_{Gln.source}$	Gln.source (ABCDE) ⟶ Gln.ext (ABCDE)
$V_{Gln.entry}$	Gln.ext (ABCDE) ⟶ Gln (ABCDE)
$V_{\alpha KGDH}$	α-kg (ABCDE) ⟶ Suc (BCDE) + CO2 (A)
$V_{Suc.release}$	Suc (ABCD) ⟶ Suc.ext (ABCD)
V_{SDH}	Suc (ABCD) ↔ Fum (ABCD)
V_{FM}	Fum (ABCD) ↔ Mal (ABCD)
V_{MDH}	Mal (ABCD) ↔ Oac (ABCD)
V_{ME}	Mal (ABCD) ↔ Pyr (ABC) + CO2 (D)
V_{AST}	Oac (ABCD) ↔ Asp (ABCD)
$V_{Asp.exch}$	Asp (ABCD) ↔ Asp.ext (ABCD)
$V_{Asp.source}$	Asp.source (ABCD) ⟶ Asp.ext (ABCD)
$V_{Mal.release}$	Mal ⟶ Mal.sink
$V_{Cit.release}$	Cit ⟶ Cit.sink
**Isotope uptake, CO_2 recycling and biomass equation**	
$V_{Gluc.tracer}$	Gluc.tracer (ABCDEF) ⟶ Gluc (ABCDEF)
$V_{Gln.tracer}$	Gln.tracer (ABCDE) ⟶ Gln.ext (ABCDE)
$V_{CO2.source}$	CO2.source (A) ⟶ CO2 (A)
$V_{CO2.sink}$	CO2 (A) ⟶ CO2.sink (A)
$V_{Biomass}$	1389*G6P ⟶ Biomass

Table S2. GC-MS fragment ions of measured metabolites regressed using the metabolic model for MFA. (Related to Fig. 4-6, S3, S5-7 and Table S1)

Metabolite	*m/z*	Derivative Formula	Carbons					
3-Phosphoglycerate	585	$C_{23}H_{54}O_7Si_4P$	**C1**	**C2**	**C3**			
Alanine	260	$C_{11}H_{26}O_2NSi_2$	**C1**	**C2**	**C3**			
Alanine	232	$C_{10}H_{26}ONSi_2$		**C2**	**C3**			
Aspartate	302	$C_{14}H_{32}O_2NSi_2$	**C1**	**C2**				
Aspartate	390	$C_{17}H_{40}O_3NSi_3$		**C2**	**C3**	**C4**		
Aspartate	418	$C_{18}H_{40}O_4NSi_3$	**C1**	**C2**	**C3**	**C4**		
Citrate	459	$C_{20}H_{39}O_6Si_3$	**C1**	**C2**	**C3**	**C4**	**C5**	**C6**
Fumarate	287	$C_{12}H_{23}O_4Si_2$	**C1**	**C2**	**C3**	**C4**		
Glucose	301	$C_{14}H_{21}O_7$	**C1**	**C2**	**C3**	**C4**	**C5**	**C6**
Glutamate	432	$C_{19}H_{42}O_4NSi_3$		**C2**	**C3**	**C4**	**C5**	
Glutamate	330	$C_{16}H_{36}O_2NSi_2$	**C1**	**C2**	**C3**	**C4**	**C5**	
Glutamine	431	$C_{19}H_{43}O_3N_2Si_3$	**C1**	**C2**	**C3**	**C4**	**C5**	
Glycerol	377	$C_{17}H_{41}O_3Si_3$	**C1**	**C2**	**C3**			
Glycerol-3-Phosphate	571	$C_{20}H_{51}O_6Si_4P$	**C1**	**C2**	**C3**			
Lactate	261	$C_{11}H_{25}O_3Si_2$	**C1**	**C2**	**C3**			
Lactate	233	$C_{10}H_{25}O_2Si_2$		**C2**	**C3**			
Malate	419	$C_{18}H_{39}O_5Si_3$	**C1**	**C2**	**C3**	**C4**		
Phosphoenolpyruvate	453	$C_{17}H_{38}O_6Si_3P$	**C1**	**C2**	**C3**			
Pyruvate	174	$C_6H_{12}O_3NSi$	**C1**	**C2**	**C3**			
Serine	390	$C_{17}H_{40}O_3NSi_3$	**C1**	**C2**	**C3**			
Succinate	289	$C_{12}H_{25}O_4Si_2$	**C1**	**C2**	**C3**	**C4**		
